# Prevalence of hepatitis B and C among female sex workers in Togo, West Africa

**DOI:** 10.1371/journal.pone.0259891

**Published:** 2021-12-10

**Authors:** Alexandra M. Bitty-Anderson, Valentine Ferré, Fifonsi A. Gbeasor-Komlanvi, Martin Kouame Tchankoni, Arnold Sadio, Mounerou Salou, Diane Descamps, Claver A. Dagnra, Charlotte Charpentier, Didier K. Ekouevi, Patrick A. Coffie

**Affiliations:** 1 Programme PACCI–Site ANRS Côte d’Ivoire, CHU de Treichville, Abidjan, Côte d’Ivoire; 2 INSERM U1219, Bordeaux Population Health Research, ISPED, Université de Bordeaux, Bordeaux, France; 3 Université de Paris, INSERM UMR 1137 IAME, Paris, France; 4 Laboratoire de Virologie, AP-HP, Hôpital Bichat-Claude Bernard, Paris, France; 5 Département de Santé Publique, Faculté des Sciences de la Santé, Université de Lomé, Lomé, Togo; 6 Centre Africain de Recherches en Epidémiologie et en Santé Publique (CARESP), Lomé, Togo; 7 Laboratoire de Biologie Moléculaire, Département des Sciences Fondamentales, Université de Lomé, Lomé, Togo; 8 Programme National de Lutte contre le VIH/Sida, les Hépatites virales et les Infections Sexuellement Transmissibles (PNLS/HV/IST), Lomé, Togo; 9 Institut de Santé Publique Epidémiologie Développement (ISPED), Université de Bordeaux, Bordeaux, France; 10 Département de Dermatologie et d’Infectiologie, UFR des Sciences Médicales, Université Félix Houphouët Boigny, Abidjan, Côte d’Ivoire; 11 CHU de Treichville, Service de Maladies Infectieuses et Tropicales, Abidjan, Côte d’Ivoire; University of Cincinnati College of Medicine, UNITED STATES

## Abstract

**Background:**

Hepatitis B and C are endemic in sub-Saharan Africa, with prevalence among the highest in the World. However, several challenges impede the progression towards the elimination of viral hepatitis by 2030 as suggested by the World Health Organization Global health sector strategy on viral hepatitis, including the lack of knowledge on the scale of this epidemic in the region. The aim of this study was to estimate the prevalence of hepatitis B and C among female sex workers (FSW) in Togo.

**Methods:**

This ancillary study from a national cross-sectional bio-behavioral study was conducted in 2017 using a respondent-driven sampling (RDS) method, in eight towns of Togo among FSW. Socio-demographic, behavioral and sexual characteristics were assessed using a standardized questionnaire. Blood samples were collected for HIV, hepatitis B and C serological testing. Data were analyzed using descriptive analysis and a logistic regression model.

**Results:**

Out of the 1,036 FSW recruited for this study, biological analyses for viral hepatitis were completed for 769 of them. The median age was 26 years [IQR: 22–33] and 49.8% (n = 383) had attained secondary school. The prevalence of hepatitis B was 9.9% [95% CI: (7.9–12.2)] and the prevalence of hepatitis C was 5.3% [95% CI: (3.9–7.2)]. Higher hepatitis B and C prevalence was associated with recruitment out of Lomé (aOR: 6.63; 95%CI: 3.51–13.40, p **<**0.001 and OR: 2.82; 95% CI: [1.37–5.99]; p**<**0.001, respectively) and, for hepatitis B, with never using condoms for vaginal intercourse (OR: 3.14; 95%CI: [1.02–8.71]; p<0.05).

**Conclusions:**

Results from this study reveals high prevalence of hepatitis B and C among FSW in Togo and an opportunity for advocacy toward the introduction of immunizations and treatment in this population.

## Introduction

Viral hepatitis is an important public health issue, with a significant burden on communities across the world [1]. It is the second major cause of death from infectious diseases after tuberculosis [2]. Viral hepatitis was estimated to be responsible for 1.4 million deaths in 2016 due to acute infection, hepatitis-related liver cancer and cirrhosis [1, 2]. Of these deaths, approximately 47% were due to hepatitis B virus (HBV), while 48% were attributable to hepatitis C virus (HCV) [1, 2]. It is estimated that 325 million people were living with HBV and HCV in 2019, with more than 80% of them lacking prevention, testing and treatment services [2]. Although mostly ignored as a public health threat until recently, viral hepatitis now figures among the targets on the United Nations 2030 Agenda for Sustainable Development [1]. The World Health Organization (WHO) Global Health Sector strategy on viral hepatitis has set a goal of eliminating viral hepatitis as a major public health threat by 2030, by reducing new infections by 90% and mortality by 65% [1, 3].

In sub-Saharan Africa (SSA), which shoulders with the Western Pacific the heaviest burden of the viral hepatitis pandemic, it is estimated that 5 to 8% of the population is affected with hepatitis B, mostly in West and Central Africa [4]. However, several barriers hinder the progression towards the goal of eliminating viral hepatitis in the African region, including the lack of action from the majority of countries with the absence of national units and allocated budgets to lead and coordinate the response [4]. In addition, the scarcity of national and subnational data impede an appropriate understanding of the public health dimension, impact and burden of the viral hepatitis pandemic as well as a difficult assessment for the allocation of resources [4]. The few available data on HBV and HCV in SSA are often focused solely on the general population, although the burden of this pandemic is potentially higher among vulnerable populations, who most often lack access to health services [5]. A study in South Africa indicated a heavy burden of HBV and HCV among key populations (people who inject drugs, sex workers and men who have sex with men) in 2018 with prevalence of hepatitis B surface antigen (HBsAg) of 4% and HCV of 16% [6]. In Nigeria, the Democratic Republic of Congo (DRC) and Rwanda, prevalence among sex workers was estimated at 17.1%, 4.20% and 2.5% respectively for HBV and 0.70% and 1.4% for HCV in the DRC and Rwanda, respectively. Another study estimated the prevalence of anti-HCV among commercial sex workers at 6.6% [7–10].

In Togo, prevalence of HBV was estimated at 7.1% among men who have sex with men (MSM) [11], while it was 10.6% among women of childbearing age [12], and 9.7% among people living with HIV (PLHIV) and treated with ART [13]. Additional data is needed to fully understand and assess the scale of the burden of hepatitis B and C among key populations, including sex workers. The aim of this study was to estimate the prevalence of HBV and HCV and its associated factors among female sex workers (FSW) in Togo in 2017.

## Materials and methods

### Study design, sampling and recruitment

This study was part of a national bio-behavioral cross-sectional study conducted from August to September 2017 in Togo. Togo is a country of West Africa, with a population of 7.6 million inhabitants in 2018, covering 57,000 square kilometres with an average density of 133 inhabitants per square kilometres, an infant mortality of 45.2/ 1,000 and an estimated life expectancy of 64.5 years old. The HIV prevalence in Togo is estimated at 2.1%, with a high prevalence among key population groups estimated between 10 and 15% [14–17]. Togo is divided into six health regions and in each region, based on the mapping and size estimation studies previously carried out in Togo [18], towns with the highest number of key populations were selected: Dapaong in the Savanes region; Kara in Kara region; Sokodé in the Centrale region; Atakpamé and Kpalimé in the Plateaux region; Tsévié and Aného in the Maritime region; and Lomé, the capital city which represents a health region. Prior to the study, locations (associations and hot spots) specific to FSW were identified during preliminary visits with the help of leaders from this community. FSW were recruited in brothels (licensed or not) and inclusion criteria were being 18 years or older, living/working/studying in Togo for a minimum of 3 months at the time of the study, being in possession of a recruitment coupon and having had sex in exchange for money as a compensation in the previous 12 months.

### Sample size estimation

The sample size estimation was based on the estimated prevalence of HBV infection among MSM in Togo estimated at 7.1% [11]. With a precision of 3% and an assumption of 10% of missing data, the minimum sample size was estimated at 348 participants.

### Data collection

After eligibility screening and written informed consent approval, trained study staff (medical students) administered a structured and standardized questionnaire during a face-to-face interview. The questionnaire was adapted from the Family Health International (FHI) 360 validated guide for bio-behavioral surveys [19] and collected information on socio-demographic characteristics, risky sexual behaviors, STIs, HIV prevention methods, HIV testing history, access to health care services, and HIV knowledge. Other validated tools were used to assess alcohol and tobacco consumption: the Alcohol Use Disorders Identification Test (AUDIT-C) and a subset of the Tobacco Questions for Surveys [20, 21]. The AUDIT-C is scored on a scale of 0–12, with scores of 0 reflecting no alcohol use. The higher the AUDIT-C score, the more likely it is that the person’s drinking is affecting his or her health. Prior to the start of the study, the questionnaire was tested among a convenient sample of 20 FSW, and 10 leaders of FSW associations across the capital, Lomé and the second most populated city, Kara.

### Biological analyses

Blood samples were collected for HIV, hepatitis B and C serological testing. HIV was assessed on site by rapid test SD Biolane Duo ® (Abbott, Santa Clara, CA, USA) and each positive result was confirmed with another HIV rapid test, the First Response® HIV 1-2-O Card Test (Premier Medical Corporation Pvt. Ltd., Maharashtra, India). In case of discordant results, samples were tested with the INNO-LIA® HIV I/II Score (20T) (Fujirebio, Göteborg, Sweden) line immunoassay. Samples were analysed for the presence of HBs antigen, HBs, HBc and HCV antibodies with ELISA technology (Alinity Abbott, Santa Clara, CA, USA).

### Statistical analysis

Descriptive statistics were performed and results were presented with frequency and proportions. Prevalence rates were estimated with their 95% confidence interval (95%CI). Continuous variables were described with median and interquartile range (IQR). Univariate and multivariate logistic regression were performed with a stepwise-descending selection procedure to identify factors associated with hepatitis B and C infection. All analyses were performed using STATA software (STATA ^TM^ 11.0 College Station, Texas, USA).

### Ethical consideration

This study was approved by the “Comité de Bioéthique pour la Recherche en Santé (CBRS)” (Bioethics Committee for Health Research) from the Togo Ministry of Health. Participants provided written consent prior to participation. Potential participants were told about the study purpose and procedures, potential risks and protections, and compensation. Informed consent was documented with signed consent forms.

## Results

### Sociodemographic characteristics and sexual practices

Out of the 1,036 FSW recruited for this study, biological analyses for hepatitis were completed for 769 of them. However, this subset of the sample was representative of the entire sample, except for HIV status as all HIV-infected participants were included in the sub-study.

The median age was 26 years (IQR: 22–33), with 62.5% of the FSW between the ages of 18 and 30. Almost half (49.8%; n = 383) achieved secondary school education and 82.4% were single. The majority reported not smoking (88.3%, n = 679) and 23% (n = 177) were at high and severe risk of harm due to alcohol consumption (AUDIT-C scores) (Table 1). The median age at first sexual intercourse was 17 years [IQR: (15–18)] and the median number of partners in the 7 days preceding the questionnaire was 7 [IQR: (4–12)]. Slightly more than half (54.2%, n = 417) reported using condoms systematically, at every sexual intercourse, for vaginal intercourse, while 4.0% (n = 31) reported never using them (Table 2). Of the 55 FSW (7.2%) who reported having been engaged in anal sex in the 7 days preceding the questionnaire, 45.5% (n = 25) reported using condoms systematically (every time). For oral intercourse, 12.4% (n = 17) of the 137 FSW who reported practicing it in the 7 days prior to the questionnaire reported systematically (every time) using condoms.

**Table 1:** Sociodemographic characteristics among female sex workers in Togo in 2017 (N = 769).

Characteristic	
**Age (years), Median (IQR)**	26 (22–33)
**Age (years)**	
18–30	521 (67.8)
30–40	183 (23.8)
≥40	65 (8.4)
**Level of education**	
None	124 (16.1)
Primary	219 (28.5)
Secondary	383 (49.8)
University	43 (5.6)
**Marital status**	
Married and living with husband/sexual partner	41 (5.4)
Married and living without husband or sexual partner	68 (8.8)
Single	634 (82.4)
No answer	26 (3.4)
**Nationality**	
Togolese	648 (84.3)
Other	121 (15.7)
**Health region**	
Centrale	58 (7.5)
Kara	71 (9.2)
Lome	412 (53.6)
Maritime	55 (7.2)
Plateaux	109 (14.2)
Savanes	64 (8.3)
**Tobacco use**	
Everyday	38 (4.9)
Less than once per day	52 (6.8)
Not at all	679 (88.3)
AUDIT- C scores*	
Low risk (0–2 points)	352 (45.8)
Moderate risk (3–5 points)	240 (31.2)
High risk (6–7 points)	94 (12.2)
Severe risk (8–12 points)	83 (10.8)

*Alcohol Use Disorders Identification Test (short version).

**Table 2:** Sexual practices among female sex workers in Togo in 2017 (N = 769).

Characteristics	
**Age at first sexual intercourse (years), Median (IQR)**	17 (15–18)
**Age at first sexual intercourse (years), n (%)**	
< 14	55 (7.2)
15–20	667 (86.7)
≥21	47 (6.1)
**Number of partners in the last 7 days, Median (IQR)**	7 (4–12)
**Number of partners in the last 7 days, n (%)**	
1–5	320 (41.6)
6–10	226 (29.4)
>10	223 (29.0)
**Anal intercourse in the last 7 days, n (%)**	55 (7.2)
**Oral intercourse in the last 7 days, n (%)**	137 (17.8)
**Use of condom for vaginal intercourse, n (%)**	
Systematically (every time)	417 (54.2)
Often	279 (36.3)
Occasionally	42 (5.5)
Never	31 (4.0)
**Use of condom for anal intercourse (n = 55), n (%)**	
Systematically (every time)	25 (45.5)
Often	9 (16.4)
Occasionally	2 (3.6)
Never	15 (27.3)
No answer	4 (7.3)
**Use of condom for oral intercourse (n = 137), n (%)**	
Systematically (every time)	17 (12.4)
Often	7 (5.1)
Occasionally	2 (1.5)
Never	111 (81.0)

### Prevalence of HBV and HCV

Of the 769 FSW tested for HBV, 76 were tested positive for the hepatitis surface antigen (HBsAg), yielding a prevalence of 9.9% [95% CI: (7.9–12.2)]. Twelve (15.8%) of them were co-infected with HIV (Table 3). The prevalence of HCV was 5.3% [95% CI: 3.9–7.2], with 41 FSW positive for HCV antibody, including 3 (7.3%) who were HIV-positive. Four FSW (0.5%) were co-infected HBV-HCV, and none had all three infections (Table 3). Among 31 FSW who reported never using condoms for vaginal intercourse, 19.4% (n = 6) were HBsAg positive, while among those that reported using condoms systematically (at every sexual intercourse) 7.4% (n = 31) were HBsAg positive.

**Table 3:** Prevalence of hepatitis B and hepatitis C among female sex workers in Togo in 2017 (N = 769).

	Overall			HIV +			HIV -			p*
	N = 769			N = 114			N = 655			
	n	%	95% CI	n	%	95% CI	n	%	95% CI	
**HBV**	76	9.9	[7.9–12.2]	12	10.5	[5.6–17.7]	64	9.8	[7.6–12.3]	0.937
**HCV**	41	5.3	[3.9–7.2]	3	2.6	[0.5–7.5]	38	5.8	[4.1–7.9]	0.244
**HBV/HCV**	4	0.5	[0.1–1.3]	0	0.0	[0.0–3.2]	4	0.6	[0.2–1.6]	0.999

### Hepatitis serological markers among HIV-positive FSW

Analyses from three HBV serological markers were performed among the 113 HIV-positive FSW: HBsAg, HBV core antibody (HBcAb/ anti-HBc), and HBV surface antibody (HBsAb/anti-HBs). Overall, 26 (23.0%) were negative for all three markers, indicating they had never been exposed to HBV and were eligible for HBV vaccination; 42 (37.2%) were only positive for HBcAb, meaning a past natural infection that could be reactivated; 12 (10.6%) were positive for HBsAg and HBcAb, indicating an acute or chronic infection and 3 of them were vaccinated for hepatitis B (2.7%). The results of this biological test is summarized in Table 4 [22].

**Table 4:** Hepatitis B serological markers profiles among HIV-positive female sex workers in Togo (n = 113†).

HBsAg^‡^	HBsAb^§^	HBcAb^¶^	Frequency	(%)	Interpretation
**─**	**─**	**─**	26	(23.0)	Never exposed to HBV infection; Eligible for HBV vaccine
**─**	**+**	**─**	3	(2.7)	Immunity due to hepatitis B vaccination
**─**	**+**	**+**	30	(26.5)	Past natural infection; cleared, immunity achieved
**─**	**─**	**+**	42	(37.2)	Past natural infection; anti-HBs has waned over time
**+**	**─**	**+**	12	(10.6)	Acute infection or chronic carrier

**─**: Negative **+**_:_ Positive.

†: One missing data.

‡: HBsAg:Hepatitis B surface Antigen.

§: HBcAb: Hepatitis B core Antibody.

¶: HBsAb: Anti-HBs (antibody to Hepatitis B surface antigen).

### Factors associated with HBV and HCV

In multivariate analysis, the recruitment region was significantly associated with HBsAg positivity (aOR = 6.63; 95%CI: 3.51–13.40, p **<**0.001) and with HCV (OR: 2.82; 95% CI: [1.37–5.99]; p **=** 0.006) so that FSW recruited from the other regions other than Lomé the capital city, were more likely to be positive for HBV and HCV. The lack of use of condoms for vaginal intercourse was also associated with HBsAg positivity (aOR = 3.14; 95% CI: 1.02–8.71), p = 0.034) (Table 5).

**Table 5:** Factors associated with hepatitis B and hepatitis C among female sex workers in Togo.

	Multivariable, Hepatitis B (n = 769)	Multivariable, Hepatitis C (n = 769)
	aOR* (95%CI, p)	aOR* (95%CI, p)
**Age (years)**		
<25	1	1
≥25	1.46 (0.86–2.52, p = 0.162)	1.28 (0.66–2.55, p = 0.470)
**Health region**		
Lomé	1	1
Other regions	6.63 (3.51–13.40, **p<0.001**)	2.82 (1.37–5.99, **p = 0.006**)
**Number of partners (previous week)**		
1–4	1	1
5–10	1.35 (0.69–2.71, p = 0.385)	1.42 (0.68–3.05, p = 0.360)
>10	1.23 (0.64–2.42, p = 0.533)	0.41 (0.15–1.03, p = 0.062)
**Anal intercourse in the last 7 days**		
No	1	
Yes	0.64 (0.17–1.86, p = 0.450)	
**Oral intercourse in the last 7 days**		
No	1	1
Yes	0.65 (0.28–1.35, p = 0.271)	0.40 (0.12–1.08, p = 0.103)
**Use of condom for vaginal intercourse**		
Systematically (every time)	1	1
Often	1.25 (0.72–2.18, p = 0.433)	1.16 (0.55–2.41, p = 0.696)
Occasionally	0.70 (0.16–2.23, p = 0.583)	1.08 (0.23–3.73, p = 0.907)
Never	3.14 (1.02–8.71, **p = 0.034**)	3.06 (0.66–10.46, p = 0.101)
**AUDIT Score**		
Low risk (0–2 points)	1	1
Moderate risk (3–5 points)	1.69 (0.93–3.09, p = 0.083)	1.65 (0.76–3.56, p = 0.199)
High risk (6–7 points)	1.45 (0.65–3.11, p = 0.344)	1.10 (0.34–3.03, p = 0.858)
Severe risk (8–12 points)	1.37 (0.53–3.25, p = 0.496)	1.92 (0.63–5.23, p = 0.222)
**Tobacco use**		
Everyday	1	
Less than once per day	1.52 (0.06–40.99, p = 0.776)	
Not at all	4.81 (0.95–87.86, p = 0.132)	
**HIV status**		
HIV -	1	1
HIV +	1.36 (0.64–2.72, p = 0.407)	0.46 (0.11–1.36, p = 0.218)
**Hepatitis C serology**		
Negative	1	NA
Positive	0.64 (0.18–1.77, p = 0.438)	NA
**Hepatitis B serology**		
Negative	NA	1
Positive	NA	0.64 (0.18–1.74, p = 0.429)

*aOR: adjusted Odds Ratio.

NA: non-applicable.

## Discussion

This study, conducted in 2017, is derived from a national study on the prevalence of HIV and hepatitis among key populations in Togo. It showed a prevalence of HBV as high as almost 10% which is comparable to previous studies among different populations in the region. In Togo, this prevalence was 9.7% among PLHIV in 2011 [13]; 7.1% (95% CI: [5.3–9.3]) among MSM [11], and 10.6% among women of childbearing age in 2017 [12]. Across the region, prevalence of HBV was approximately similar in Côte d’Ivoire, Burkina Faso and Mali with a prevalence of 8.5% among PLHIV [23]. Among FSW, a study in South African found a prevalence of 4% in 2017 and another one in Kenya a prevalence of 10.1% in 2020 [6, 24]. Those results demonstrate that HBV remains endemic in sub-Saharan Africa, despite the framework for action for viral hepatitis in the African region aiming for the elimination of viral hepatitis as a major public health threat by 2030 [4].

The findings from this study also indicates that the question of immunization against HBV remains. In this study, only 3% of HIV-positive FSW were vaccinated against HBV. For people living with HIV (PLHIV), immunity to HBV often necessitates a double dose of the vaccine, which could further reduce the actual proportion of FSW that are indeed immune to HBV [25, 26]. In addition, 23% had never been exposed to HBV and needed to be vaccinated against HBV. No other study was found on the immunization status of FSW in Lomé and in West Africa; however, a study conducted among mothers in Lomé in 2017 reported that 65.2% of 89 women were not vaccinated [12]. Moreover, in Togo, HBV immunization is part of the Expanded Program on Immunization (EPI), is required free of charge as part of this program for children less than 12 months old specifically at 6, 10 and 14 weeks of life, and has been introduced only in 2008 [12]; apart from the EPI, HBV vaccination is available at approximately 5 euros. Data from America and Europe suggest rates of HBV immunization of FSW are higher than in sub-Saharan Africa. In a study in Brazil, HBV vaccination rate was indeed 27.6% among 721 FSW and of the 434 eligible for vaccination, 89.6% accepted the first HBV vaccine dose, and 37.5% completed the 3 doses of the vaccine [27]. In Vancouver (Canada), 68.3% reported lifetime HBV vaccination [28] and approximately the same rate was reported in the Netherlands (63%) among sex workers [29]. The American College of Physicians also advises as a primary best practice for HBV prevention the vaccination against HBV in all unvaccinated adults at risk of infection (due to sexual, percutaneous, or mucosal exposure; health care and public safety workers at risk of blood exposure; adults with chronic liver diseases, end-stage renal infection, HIV infection) [30]. In this sense, additional efforts and strategies should be geared towards adults’ screening and vaccinations, including high risk individuals such as FSW.

HBV serological markers test performed on HIV-positive FSW confirmed that the majority of HBsAg-positive FSW included in the analysis are chronic carriers as transmission mostly occurs during childhood in sub-Saharan Africa [2] and thus, could potentially be eligible for treatment. The WHO defines treatment eligibility as the presence of fibrosis with the APRI score >2 (aspartate aminotransferase [AST]-to-platelet ratio index) in adults aged 30 years or older, persistently abnormal ALT (Alanine aminotransferase) levels, and high HBV replication (HBV DNA>20 000 IU/m L) [31]. A study conducted in Côte d’Ivoire, Togo and Burkina Faso among inmates, MSM and FSW in 2017 indicated that with a prevalence of hepatitis B infection of 10.6%, 11% were eligible for treatment, as per WHO eligibility criteria [32]. Hence, in this study, there could be an approximate similar proportion of FSW eligible for treatment.

In terms of treatment, the WHO recommends the use of Tenofovir or Entecavir as the most potent drugs for hepatitis B virus, with the cost of WHO prequalified generic Tenofovir estimated at USD 32 per year [33]. However, in Togo, although treatment is available, there is a lack of hepatitis B national treatment programs for patients that would subsidize treatment costs for patients unable to afford them. With the absence of universal health insurance, FSW specifically those HBV mono-infected (since those that are HIV co-infected have access to treatment through HIV care and treatment programs) would hardly be able to afford this lifelong treatment as over 50% of the population live below the poverty line (under 1.03 euros per day) [34]. One study conducted in Togo among chronic HBV patients indicated that the overall cost for pre-therapeutic assessment for chronic viral hepatitis (examinations to evaluate fibrosis, biochemical, biological, immunological, virological and radiological examinations) and the actual treatment with Tenofovir within a 13-month period averaged 501.3 euros for uninsured patients, 499.1 euros for patients with official health insurance and 261.6 euros for patients with private health insurance [35]. Advocacy for funding of the national program against HIV, STI and viral hepatitis specifically for treatment and care of hepatitis B among chronic HBV patients could be a step toward prevention with the reduction of transmission and the prevention of the serious consequences of HBV (liver cancer, cirrhosis, etc), hence a potential reduction of an additional financial burden on the health care system.

Our study showed that more than a quarter of FSW never used condoms and were approximately three times more likely to be infected with HBV. A similar result was found in a study in Thailand among migrant sex workers [36]. This subgroup of FSW who do not systematically use condoms with multiple sex partners is where immunization strategies should be focused on. Outreach programs and awareness campaigns specifically designed for FSW on viral hepatitis could potentially have an impact both on condom use and HBV screening and immunization uptake.

In our study, prevalence of HBV and HCV was higher among FSW recruited in regions other than Lomé. This trend is however different from what is observed with the HIV epidemic in Togo with higher prevalence of HIV in Lomé compared to other regions. The fact that HBV prevalence was higher among regions other than Lomé was confirmed by another study in Togo conducted among the general population which found membership into the Kabyè-tem ethnic group from the central and north regions of Togo as an independent risk factor for HBsAg positivity [37]. To our knowledge, no studies have explored the reasons for this correlation. However, hypotheses include genetic or cultural factors [37]. Further studies should specifically explore this aspect of the viral hepatitis pandemic in other regions of Togo to identify specific ways to prevent transmission.

Very few studies in Togo have explored hepatitis C prevalence. A study among 1,213 adults in Lomé found a 5.6% prevalence of HCV in 2011 [38]. Our study found a prevalence of 5.3% which is approximately similar to the rates found in sub-Saharan Africa. A systematic review found an HCV prevalence of 4.14% in Western Africa in 2016 [39] and of 6.5% in Cameroon [40] based solely on the presence of HCV antibodies. Other studies in the region found HCV prevalence of 2.6% in 2015 in sub-Saharan Africa [41], and of 3% in Ghana in 2016, with results from viral load testing [40, 42]. Another study among HIV-1, HIV-2 and dually reactive patients in West Africa found an anti-HCV prevalence of 3.9% and a HCV RNA prevalence of 2.7% [43]. Overall, despite HCV prevalence estimates being lower than that of HBV, it remains a cause of concern. As is the case with hepatitis B, there is no access to treatment for HCV patients through the national program or a treatment program for HCV. The results from this study could play a role as a stepping stone toward promoting access to HCV care and treatment.

Our study presents some limitations including the fact that all HBV serologic markers were only performed on HIV-positive FSW due to limited funds available for this supplementary analysis on hepatitis B. For hepatitis B, we also did not perform HBV DNA level to determine eligibility to HBV treatment. However, a study in West Africa indicated that 11% of HBV chronic carriers were eligible for HBV treatment; hence we can assume that a similar proportion of FSW in our sample would be eligible for HBV treatment [32]. For HCV, we were not able to perform viral load tests nor a second test after 6 months due to insufficient amounts of serums. Therefore, HCV prevalence was probably overestimated in our sample. Also, the aspartate aminotransferase to platelet ratio index (APRI) score (an index recommended by the WHO for the assessment of liver fibrosis by non-invasive tests that estimate hepatic fibrosis) [31] was not determined and genotypic tests could not be completed as well for both hepatitis B and C; however, it has been determined that genotype E is the predominant genotype in West Africa for HBV and genotypes 1 and 2 for HCV [32, 44]. Finally, we did not collect information on at-risk practices, such as syringes exchanges in drug users; however, this practice is very limited in West Africa [45]. Nevertheless, results from this study are among the first in the country and in the West African region to explore both hepatitis B and C among FSW, a vulnerable population, in order to fill the gap on data of viral hepatitis in Western Africa among key populations. It is also among the first studies to document hepatitis B serological markers among FSW in order to assess their immunization status, even though it was done among HIV-positive FSW only. In addition, this study was conducted among a large sample of FSW and across the 6 health regions of the country, hence a high external validity making it generalizable to FSW of other countries of West Africa.

## Conclusions

This study confirms a high prevalence HBV and HCV among FSW, suggesting the importance of testing, including partners’ testing specifically for HBV prevention, especially since the use of condoms in this population is not systematic. There is a need for viral hepatitis to be integrated into national health systems and strategies, with for example an integrated approach to the package of testing and care for key populations, that also includes HIV and sexually transmitted infections. Hepatitis surveillance studies and not only for HIV are needed, as well as additional and adequate data on the impact of the hepatitis pandemic that would reinforce the advocacy for national viral hepatitis strategies, plans and allocated budgets. Further studies should also explore the regional differences in viral hepatitis prevalence between the North and the South of Togo.

## Supporting information

S1 File Questionnaire original version (Français). (PDF)

S2 File Questionnaire English version. (PDF)

S1 Data (XLSX)
