# Supplementary material for: Prevalence of hepatitis B and C among female sex workers in Togo, West Africa
Source: PLoS One. 2021 Dec 10;16(12):e0259891. doi: 10.1371/journal.pone.0259891 (PMC8664183; doi:10.1371/journal.pone.0259891)
Supplement: S1 File — (PDF) [file pone.0259891.s001.pdf]

Questionnaire PS, Togo 2017

Nom de la ville : ____

Identifiant: ____

Date de l'entretien/ou du recueil des données: _____ / _____ / 2017

Enquêteur: ______________________________ [code : ....]

Section 1 : Caractéristiques sociodémographiques

N°	Questions et renvois	Réponses codées	Saut
101	Quel âge avez-vous ?<br>***Arrêt de l'entretien si âge inférieur à 18 ans ou né après Août 1999.***	|___|___|/|___|___|/|___|___|___|___| Jour Mois Année<br>|__|__| Age en années révolues<br>88. Ne sait pas<br>99. Pas de réponse	|____|
102	Avez-vous été à l'école ?	0. Non →<br>1. Oui<br>99. Pas de réponse	104<br>|____|
103	Quel niveau d'éducation avez-vous atteint ?<br>***ENTOUREZ UNE SEULE REPONSE.***	1. Primaire<br>2. Secondaire<br>3. Supérieur<br>99. Pas de réponse	|____|
104	Quelle est votre religion ?<br>***ENTOUREZ UNE SEULE REPONSE.***	0. Non croyant<br>1. Animiste<br>2. Chrétien<br>4. Musulman<br>5. Autres, précisez : ___________________________	|____|
105	Quelle est votre nationalité ?	1. Togolaise<br>2. Autre, précisez : ___________________________	|____|
106	Quelle est votre ethnie ?<br>***ENTOUREZ UNE SEULE REPONSE.***	1. Adja-Ewe<br>2. Akposso<br>3. Ana-Ife<br>4. Kabyè<br>5. Kotokoli<br>6. Mina<br>7. Moba<br>6. Tchamba<br>8. Autres, Précisez : ___________________________	|____|
107	Avez-vous déjà été mariée ?	0. Non<br>1. Oui<br>99. Pas de réponse	|____|
108	Quelle est votre situation matrimoniale actuelle ?	1. Mariée et vivant avec époux<br>2. Mariée et vivant avec un autre partenaire sexuel<br>3. Mariée ne vivant ni avec époux ni avec un autre partenaire sexuel<br>4. Non mariée et vivant avec un partenaire sexuel<br>5. Non mariée et ne vivant pas avec un partenaire sexuel<br>99. Pas de réponse	→110
109	Votre mari (ou votre partenaire) a-t-il plus d'une épouse / partenaire ?	0. Non<br>1. Oui<br>88. Ne sait pas<br>99. Pas de réponse	|____|
110	Combien avez-vous d'enfants en tout ?<br>(vos propres enfants)	|__|__| enfants<br>***MARQUEZ 00 SI PAS D'ENFANTS.***	

Section 1 : Caractéristiques sociodémographiques (suite)

**111**	A quel âge avez-vous commencé à gagner de l'argent en proposant des rapports sexuels rémunérés ?	|__|__| Age en années<br>88. Ne sait pas<br>99. Pas de réponse	|__|
**112**	Avez-vous d'autres activités vous rapportant des revenus complémentaires ?	0. Non → **114**<br>1. Oui<br>99. Pas de réponse	|__|
**113**	Quelles sont ces activités ?<br><br>***REPONSES MULTIPLES POSSIBLES.***	1. Cadre dans une structure privée 0. Non 1. Oui<br>2. Cadre dans une structure publique 0. Non 1. Oui<br>3. Ventes 0. Non 1. Oui<br>4. Services (coiffeuse, couturière…) 0. Non 1. Oui<br>5. Travail manuel non qualifié 0. Non 1. Oui<br>6. Travail manuel qualifié 0. Non 1. Oui<br>7. Employée de maison 0. Non 1. Oui<br>8. Travail agricole 0. Non 1. Oui<br>9. Autres, 0. Non 1. Oui<br>précisez : ____________________	|__| |__| |__| |__| |__| |__| |__| |__| |__|
**114**	Etes-vous le soutien financier de votre famille (enfants, parents) ou le soutien financier d'autres personnes ?	0. Non<br>1. Oui<br>99. Pas de réponse	|__|
**115**	Combien de personnes dépendent actuellement de vos revenus ?	|__|__| personnes<br>88. Ne sait pas<br>99. Pas de réponse	|__|

Section 2 : Antécédents sexuels ; nombre et types de partenaires

**J'aimerais maintenant vous poser quelques questions sur vos partenaires sexuels.**			
**201**	A quel âge avez-vous eu votre premier rapport sexuel ?	|__|__| Age en années<br>88. Ne sait pas<br>99. Pas de réponse	|__|
**202**	Parmi l'ensemble de vos partenaires sexuels de ces 7 derniers jours, combien étaient…<br>- des CLIENTS (partenaires avec lesquels vous avez eu un rapport sexuel en échange d'argent) ?<br><br>- des AUTRES PARTENAIRES (ceux avec lesquels vous avez des rapports sexuels, mais pas pour de l'argent) ?<br>***INCLUEZ DANS CETTE DERNIERE CATEGORIE L'EPOUX OU LE(S) CONCUBIN(S).***	|__|__| **CLIENTS**<br>88. Ne sait pas<br>99. Pas de réponse<br><br>|__|__| **AUTRES PARTENAIRES**<br>88. Ne sait pas<br>99. Pas de réponse	|__| <br><br><br>|__|
**203**	Au total, combien de partenaires sexuels différents avez-vous eu durant les sept (7) derniers jours ?<br><br>***INCLUEZ EPOUX ET CONCUBINS. VERIFIEZ QUE LES NOMBRES TOTAUX OBTENUS AVEC LA Q202 ET AVEC LA Q203 SONT EGAUX.***	|__|__| **NOMBRE TOTAL de PARTENAIRES**<br>88. Ne sait pas<br>99. Pas de réponse	|__|

Section 3 : Rapports sexuels payants

**301**	Combien de clients avez-vous eu durant votre dernière journée de travail ?	|__|__| **NOMBRE DE CLIENTS**<br>88. Ne sait pas<br>99. Pas de réponse	|____|
**302**	Combien d'argent avez-vous gagné avec votre dernier client ?	|__|__|__|__|__|__|__| Francs CFA<br>88. Ne sait pas<br>99. Pas de réponse	|____|

Section 4 : Rapports sexuels avec autres partenaires

	**RENVOI : VOIR la question 202**<br>A UN (DES) PARTENAIRE(S) SEXUEL(S) AUTRES QUE SES CLIENTS |__| ↓	N'A PAS DE PARTENAIRE SEXUEL AUTRE QUE SES CLIENTS |__| → **501**	
**401**	Essayez de vous rappeler la dernière personne avec laquelle vous avez eu un rapport sexuel non payant. Combien de rapports avez-vous eu avec cette personne au cours des trente (30) derniers jours ?	|__|__| Nombre de fois<br>88. Ne sait pas<br>99. Pas de réponse	|____|

Section 5 : Préservatifs

**Préservatifs masculins : Connaissances et disponibilité**			
**501**	Avez-vous déjà entendu parler du préservatif masculin ?<br>***MONTREZ SPECIMEN OU PHOTO.***	0. Non → **503**<br>1. Oui<br>88. Ne sait pas<br>99. Pas de réponse	|____|
**502**	Avez-vous déjà utilisé un préservatif masculin avec un **quelconque** de vos partenaires sexuels ?	0. Non → **503**<br>1. Oui<br>88. Ne sait pas<br>99. Pas de réponse	|____|
**503**	Connaissez-vous un endroit ou une personne qui peut vous fournir en préservatifs masculins ?<br>***ENTOUREZ UNE SEULE REPONSE.***	0. Non → **506**<br>1. Oui<br>99. Pas de réponse → **506**	|____|
**504**	Dans quels endroits ou auprès de quelles personnes pouvez-vous vous procurer des préservatifs masculins ?<br>***REPETEZ LA QUESTION ET MARQUEZ TOUTES LES REPONSES DONNEES.***	a. Boutique 0. Non 1. Oui<br>b. Pharmacie 0. Non 1. Oui<br>c. Marché 0. Non 1. Oui<br>d. Hôpital 0. Non 1. Oui<br>e. Dispensaire 0. Non 1. Oui<br>f. Centre de planning familial 0. Non 1. Oui<br>g. Bar/ Hôtel 0. Non 1. Oui<br>h. Jeune éducateur 0. Non 1. Oui<br>i. Ami(e) 0. Non 1. Oui	|____
____
____
____
____
____
____
____
____	

		j. Autre 0. Non 1. Oui<br>Précisez : ________________________<br>99. Pas de réponse 0. Non 1. Oui	|____| |____| |____|
505	Combien de temps vous faut-il pour vous procurer un préservatif masculin depuis votre domicile ou votre lieu de travail ?	1. Moins d'une heure<br>2. Entre une heure et un jour<br>3. Plus d'un jour<br>88. Ne sait pas<br>99. Pas de réponse	|____|
506	Comment vous procurez-vous des préservatifs masculins ?	1. Par achat<br>2. Gratuitement<br>3. Par achat et gratuitement	|____|
507	Combien vous coûte l'achat de préservatifs masculins par mois ?	|___|___|___|___|___| Francs CFA<br>97. Non applicable<br>88. Ne sait pas	|____|
508	Combien de préservatifs masculins avez-vous dans votre chambre et/ou sur vous ?	|__|__|__| préservatifs disponibles<br>99. Pas de réponse	|____|

**Niveau de confiance en la protection par le préservatif**			
**509**	Selon vous, l'utilisation du préservatif protège contre la survenue de l'infection à VIH	1. Oui entre 80% et 100%<br>2. Oui mais entre 60% et 80%<br>3. Oui à 50%<br>4. Non, ne protège pas<br>99. Ne sait pas	|____|
**Préservatif et type de rapport sexuel**			
**510**	Quel est le type de rapport sexuel que vous avez eu ?<br>*PLUSIEURS REPONSES POSSIBLES.*	1. Oral<br>2. Vaginal<br>3. Anal	|____|
**511**	Avez-vous utilisé un préservatif pour ce rapport sexuel oral (fellation, anulingus, cunilingus)?	1. Systématiquement<br>2. Souvent<br>3. Occasionnellement<br>4. Jamais<br>5. Non applicable	
**512**	Avez-vous utilisé un préservatif pour ce rapport sexuel anal ?	1. Systématiquement<br>2. Souvent<br>3. Occasionnellement<br>4. Jamais<br>5. Non applicable	|____|
**513**	Utilisez-vous un préservatif pour un rapport vaginal ?	1. Systématiquement<br>2. Souvent<br>3. Occasionnellement<br>4. Jamais	|____|
**514**	Quel était ce partenaire ?	1. Un partenaire connu<br>2. Un nouveau partenaire<br>3. Un inconnu<br>4. Un client ou cliente	
**Préservatif et dernier rapport sexuel**			
**513**	Avez-vous utilisé un préservatif au cours du dernier rapport sexuel ?	0. Non → **514**<br>1. Oui<br>88. Ne sait pas } → **515**<br>99. Pas de réponse	|____|

**514**	Pourquoi n'avez-vous pas utilisé de préservatif ?	1. Refus du partenaire<br>2. Préservatif non disponible<br>3. Je connais le statut du partenaire<br>4. Autres, préciser : ___________________________	|___|
**515**			
**516**			
**Préservatif et dernier rapport sexuel avec un client**			
**517**	Lors de votre dernier rapport sexuel avec un client, avez-vous utilisé un préservatif ?	0. Non → **519**<br>1. Oui<br>88. Ne sait pas } → **518** → **306**<br>99.Pas de réponse	|___|
**518**	Qui a proposé l'emploi d'un préservatif durant ce rapport ?<br><br>***ENTOUREZ UNE SEULE REPONSE.***	1. Moi-même<br>2.Partenaire<br>3. Décision commun<br>88. Ne sait pas<br>99.Pas de réponse } → **520**	|___|
**519**	Pourquoi n'avez-vous pas utilisé de préservatif pour ce rapport ?<br><br>***ENTOUREZ TOUTES LES REPONSES DONNEES.***	a. Pas de préservatif disponibleo. Non 1. Oui<br>b. Préservatif trop cher 0. Non 1. Oui<br>c. Objection du partenaire 0. Non 1. Oui<br>d. N'aime pas le préservatif 0. Non 1. Oui<br>e. A utilisé un autre contracepto. Non 1. Oui<br>f. A jugé le préservatif inutile 0. Non 1. Oui<br>g. N'y a pas pens 0. Non 1. Oui<br>h. Autre 0. Non 1. Oui<br>Précisez:___________________________________<br>88. Ne sait pas<br>99. Pas de réponse	|___| |___| |___| _<br>|___| |___| |___| |___| |___| |___|
**520**	Durant les trente (30) derniers jours, avez-vous fréquemment utilisé un préservatif avec vos **clients** ?	1. Chaque fois<br>2. Souvent<br>3. Rarement<br>4. Jamais<br>88. Ne sait pas<br>99. Pas de réponse	|___|
**Préservatifs et rapports sexuels avec autres partenaires**			
**521**	Lors de votre dernier rapport sexuel avec un autre partenaire, avez-vous utilisé un préservatif ?	0. Non → **523**<br>1. Oui<br>88. Ne sait pas<br>99.Pas de réponse	|___|
**522**	Qui a proposé l'emploi d'un préservatif durant ce rapport ?<br><br>***ENTOUREZ UNE SEULE REPONSE.***	1. Moi-même mmême<br>2.Partenaire PpPartenaire<br>3. Décision commune<br>88. Ne sait pas<br>99.Pas de réponse } → **524**	|___|
**523**	Pourquoi n'avez-vous pas utilisé de préservatif pour ce rapport ?	a. Pas de préservatif disponibleo. Non 1. Oui<br>b. Préservatif trop cher 0. Non 1. Oui	|___| |___|

	ENTOUREZ TOUTES LES REPONSES DONNEES.	c. Objection du partenaire 0. Non 1. Oui<br>d. N'aime pas le préservatif 0. Non 1. Oui<br>e. A utilisé un autre contracept0. Non 1. Oui<br>f. A jugé le préservatif inutile 0. Non 1. Oui<br>g. N'y a pas pens 0. Non 1. Oui<br>h. Autre 0. Non 1. Oui<br>Précisez:________________________________<br>88. Ne sait pas<br>99. Pas de réponse	|____| _<br>|____| |____| |____| |____| |____| |____|
**524**	Durant les trente (30) derniers jours, avez-vous fréquemment utilisé un préservatif avec vos autres partenaires?	1. Chaque fois<br>2. Souvent<br>3. Rarement<br>4. Jamais<br>88. Ne sait pas<br>99. Pas de réponse	|____|
**Dernier rapport sexuel sans préservatif**			
**525**	A quand remonte votre dernier rapport sexuel sans utilisation de préservatif ?	0. Dernière nuit<br>1. Moins d'une semaine<br>2. Moins d'un mois<br>3. Moins de six mois<br>4. Je ne me souviens pas	|____|
**526**	Pourquoi n'avez-vous pas utilisé de préservatif ?	1. Refus du partenaire<br>2. Préservatif non disponible<br>3. Je connais le statut du partenaire<br>4. Autres, préciser : ________________________	|____|
**527**	Quel est le type de rapport sexuel que vous avez eu ?	1. Oral<br>2. Vaginal<br>3. Anal	|____|
**528**	Quel était ce partenaire ?<br><br>*PLUSIEURS REPONSES POSSIBLES.*	1. Un partenaire connu<br>2. Un nouveau partenaire<br>3. Un inconnu<br>4. Un client ou cliente	|____|

**Circonstances de rapport sexuel sans préservatif**			
**529**	Dans quelles circonstances accepteriez-vous d'avoir un rapport sexuel sans préservatif ?	0. Jamais<br>1. Rapports forcés<br>2. Pour de l'argent<br>3. Avec un partenaire que je connais bien<br>4. Je connais le statut VIH du partenaire<br>5. Autres, préciser : ___________________________	|____|
**Accident au préservatif**			
**530**	Arrive-t-il que les préservatifs que vous utilisez se déchirent lors de vos rapports sexuels?	0. Non, jamais<br>1. Oui, rarement<br>2. Oui, souvent<br>3. Oui, très fréquemment	|____|
**Préservatifs féminins**			
**531**	Avez-vous déjà entendu parler du préservatif féminin ?	0. Non<br>1. Oui<br>88. Ne sait pas<br>99. Pas de réponse	|____|

	MONTREZ UN SPECIMEN OU UNE PHOTO.		
**532**	Avez-vous déjà utilisé le préservatif féminin ?<br><br>***MONTREZ UN SPECIMEN OU UNE PHOTO.***	0. Non<br>1. Oui<br>2. Non applicable<br>88. Ne sait pas<br>99. Pas de réponse	|___|
**533**	Connaissez-vous un endroit ou une personne qui peut vous fournir en préservatifs féminins ?	0. Non<br>1. Oui<br>2. Non applicable<br>99. Pas de réponse	|___|
**534**	Dans quels endroits ou auprès de quelles personnes pouvez-vous vous procurer des préservatifs féminins ?<br><br>***REPETEZ LA QUESTION ET MARQUEZ TOUTES LES REPONSES DONNEES.***	a. Boutique 0. Non 1. Oui<br>b. Pharmacie 0. Non 1. Oui<br>c. Marché 0. Non 1. Oui<br>d. Hôpital 0. Non 1. Oui<br>e. Dispensaire 0. Non 1. Oui<br>f. Centre de planning familial 0. Non 1. Oui<br>g. Bar / Hôtel 0. Non 1. Oui<br>h. Jeune éducateur 0. Non 1. Oui<br>i. Ami(e) 0. Non 1. Oui<br>h. Autre 0. Non 1. Oui<br>Précisez :________________<br>i. Non applicable 0. Non 1. Oui<br>99. Pas de réponse 0. Non 1. Oui	|___| |___| |___| |___| |___| |___| |___| |___| |___| |___| <br>|___| |___|

Section 6 : IST

**601**	Avez-vous déjà entendu parler de maladies qui peuvent se transmettre durant un rapport sexuel appelées infections sexuellement transmissibles (IST) ?	0. Non<br>1. Oui<br>99. Pas de réponse	|___|
**602**	Connaissez-vous certains symptômes des IST chez la femme ?<br>En connaissez-vous d'autres ?<br><br>***NE LISEZ PAS LES SYMPTOMES.***<br><br>***ENTOUREZ (1) POUR CHAQUE SYMPTOME MENTIONNE.***<br><br>***ENTOUREZ (0) POUR TOUT SYMPTOME NON MENTIONNE.***<br><br>***REPONSES MULTIPLES POSSIBLES.***	a. Douleurs abdominales 0. Non 1. Oui<br>b. Pertes malodorantes 0. Non 1. Oui<br>c. Pertes vaginales 0. Non 1. Oui<br>d. Démangeaisons 0. Non 1. Oui<br>e. Miction douloureuse 0. Non 1. Oui<br>f. Lésions génitales 0. Non 1. Oui<br>g. Gonflements dans l'aine 0. Non 1. Oui<br>h. Autres, 0. Non 1. Oui<br>Précisez : ________________<br>99. Pas de réponse 0. Non 1. Oui	|___| |___| |___| |___| |___| |___| |___| |___| <br>|___|
**603**	Connaissez-vous certains symptômes des IST chez l'homme ?<br>En connaissez-vous d'autres ?<br><br>***NE LISEZ PAS LES SYMPTOMES.***<br><br>***ENTOUREZ (1) POUR CHAQUE SYMPTOME MENTIONNE.***	a. Ecoulement urétral 0. Non 1. Oui<br>b. Gonflements dans l'aine 0. Non 1. Oui<br>c. Miction douloureuse 0. Non 1. Oui<br>d. Démangeaisons 0. Non 1. Oui<br>e. Autre 0. Non 1. Oui<br>Précisez : ________________	|___| |___| |___| |___| |___|

	ENTOUREZ (0) POUR TOUT SYMPTOME NON MENTIONNE.<br><br>***REPONSES MULTIPLES POSSIBLES.***	99. Pas de réponse 0. Non 1. Oui	|____|
**604**	Avez-vous eu des **pertes vaginales** durant les douze (12) derniers mois ?	0. Non<br>1. Oui<br>88. Ne sait pas<br>99. Pas de réponse	|____|
**605**	Avez-vous eu des **ulcérations/ lésions** dans la zone génitale durant les douze (12) derniers mois ?	0. Non<br>1. Oui<br>88. Ne sait pas<br>99. Pas de réponse	|____|
**606**	La dernière fois que vous avez eu des pertes/écoulements ou une ulcération/lésion génitale, avez-vous ?	NON OUI Ne sait pas Pas de réponse	
	a. consulté dans un dispensaire ou un hôpital pour obtenir conseils et/ou médicaments ?	0 1 88 99	|____|
	b. consulté dans une pharmacie pour obtenir conseils et/ou médicaments ?	0 1 88 99	|____|
	c. consulté un guérisseur traditionnel pour obtenir conseils et/ou médicaments ?	0 1 88 99	|____|
	d. pris des médicaments que vous aviez déjà chez vous ?	0 1 88 99	|____|
	e. discuté avec votre partenaire sexuel(le) de ces écoulements ou de ces lésions (IST) ?	0 1 88 99	|____|
	f. cessé tout rapport sexuel tant que les symptômes existaient ?	0 1 88 99	|____|
	j. utilisé un préservatif durant vos rapports sexuels tant que les symptômes ont persisté ?<br><br>***LISEZ LES ENONCES SUIVANTS. REPONSES MULTIPLES POSSIBLES.***	0 1 88 99	|____|
**607**	Quelle a été votre première démarche ?<br><br>***UNE SEULE REPONSE POSSIBLE.***	1. Consultation dans un dispensaire/hôpital pour obtenir conseils et/ou médicaments ?<br>2. Consultation dans une pharmacie pour obtenir conseils et/ou médicaments ?<br>3. Consultation d'un guérisseur traditionnel pour obtenir conseils et/ou médicaments ?<br>4. Prise de médicaments que vous aviez chez vous ?<br>5. Autre, précisez : ____________________<br>88. Ne se souvient pas<br>99. Pas de réponse	|____|
**608**	Si vous avez pris des médicaments la dernière fois que les symptômes sont apparus, qui vous les a procurés ?<br><br>***ENTOUREZ TOUTES LES REPONSES DONNEES.***	a. Dispensaire 0. Non 1. Oui<br>b. Pharmacie 0. Non 1. Oui<br>c. Guérisseur traditionnel 0. Non 1. Oui<br>d. Ami(e) ou parent 0. Non 1. Oui<br>e. Médicament chez soi 0. Non 1. Oui<br>f. N'a pas pris de médicaments 0. Non 1. Oui<br>88. Ne se souvient pas 0. Non 1. Oui<br>99. Pas de réponse 0. Non 1. Oui	|____| |____| |____| |____| |____| |____| |____| |____|
**609**	Combien avez-vous payé pour vos médicaments ?	|___|___|___| ___|___|___| Francs CFA	
**610**	**RENVOI : VOIR les questions 606, 607, 608**<br>A consulté un agent de santé dans un dispensaire ou un hôpital	0. Non **→ 701**<br>1. Oui	|____|
**611**	Combien de temps avez-vous attendu entre la première apparition des symptômes et votre visite dans un dispensaire ou un hôpital ?	1. 1 semaine ou moins<br>2. Entre une semaine et un mois<br>3. 1 mois ou plus<br>88. Ne sait pas<br>99. Pas de réponse	|____|

612	Vous a-t-on délivré une ordonnance pour vos médicaments ?	0. Non<br>1. Oui<br>88. Ne sait pas<br>99. Pas de réponse	|___|
613	Avez-vous pu obtenir les médicaments prescrits ?	0. Non<br>1. Oui } → 701<br>3. En partie seulement<br>88. Ne se souvient pas<br>99. Pas de réponse	|___|
614	Si vous n'avez pas pris tous les médicaments prescrits, pour quelle(s)raison(s) ?<br><br>***ENTOUREZ TOUTES LES REPONSES DONNEES.***	a. Pas d'argent pour les acheter 0. Non 1. Oui<br>b. Médicaments non disponibles 0. Non 1. Oui<br>c. N'aime pas prendre les médicaments 0. Non 1. Oui<br>d. Autres 0. Non 1. Oui<br>Précisez : ________________<br>88. Ne se souvient pas<br>99. Pas de réponse	|___| |___| |___| |___| |___|

Section 7 : Connaissances et attitudes à propos du VIH / sida

701	Avez-vous déjà entendu parler du VIH ou d'une maladie qui s'appelle le sida ?	0. Non → 801<br>1. Oui<br>99. Pas de réponse	|___|
702	Connaissez-vous quelqu'un qui est infecté par le VIH ou qui est mort du sida ?	0. Non → 704<br>1. Oui<br>88. Ne sait pas<br>99. Pas de réponse → 704	|___|
703	Avez-vous un(e) parent(e) ou un(e) ami(e)proche qui est infecté(e)par le VIH ou qui est mort(e) du sida ?	0. Non<br>1. Oui<br>88. Ne sait pas<br>99. Pas de réponse	|___|
704	Peut-on se protéger contre le VIH, le virus qui cause le sida, en utilisant un préservatif de manière correcte et systématique (à chaque rapport sexuel) ?	0. Non<br>1. Oui<br>88. Ne sait pas<br>99. Pas de réponse	|___|
705	Peut-on être contaminé(e) avec le VIH par une piqûre de moustique ?	0. Non<br>1. Oui<br>88. Ne sait pas<br>99. Pas de réponse	|___|
706	Peut-on se protéger contre le VIH en ayant des rapports sexuels exclusivement avec une personne qui est à la fois fidèle et non infectée ?	0. Non<br>1. Oui<br>88. Ne sait pas<br>99. Pas de réponse	|___|
707	Peut-on se protéger contre le VIH en s'abstenant de tout rapport sexuel ?	0. Non<br>1. Oui<br>88. Ne sait pas<br>99. Pas de réponse	|___|
708	Peut-on être contaminé(e) par le VIH en partageant un repas avec une personne infectée ?	0. Non<br>1. Oui<br>88. Ne sait pas<br>99. Pas de réponse	|___|

**709**	Peut-on être contaminé(e)par le VIH en partageant une aiguille hypodermique déjà utilisée par quelqu'un d'autre ?	0. Non<br>1. Oui<br>88. Ne sait pas<br>99. Pas de réponse	|____|
**710**	Pensez-vous qu'une personne paraissant en bonne santé peut en fait être infectée par le VIH, le virus qui cause le sida ?	0. Non<br>1. Oui<br>88. Ne sait pas<br>99. Pas de réponse	|____|
**711**	Si une femme enceinte a le VIH ou le sida, peut-elle transmettre le virus à l'enfant qu'elle porte ?	0. Non **→ 713**<br>1. Oui<br>88. Ne sait pas<br>99. Pas de réponse **→ 713**	|____|
**712**	Que peut faire une femme enceinte pour réduire le risque de transmission du VIH à l'enfant qu'elle porte ?<br>***NE LISEZ PAS LES REPONSES. ENTOUREZ TOUTES LES REPONSES DONNEES.***	a. Traitement antirétroviral 0. Non 1. Oui<br>b. Autres 0. Non 1. Oui<br>Précisez : ____________________<br>88. Ne sait pas<br>99. Pas de réponse	|____| |____| |____|
**713**	Si une femme a le VIH ou le sida, peut-elle transmettre le virus à son bébé quand elle l'allaite au sein ?	0. Non<br>1. Oui<br>88. Ne sait pas<br>99. Pas de réponse	|____|
**714**	Est-il possible dans votre communauté de passer un test confidentiel pour savoir si l'on est infecté par le VIH ?<br>***Par confidentiel, je veux dire que personne ne peut savoir le résultat du test si vous préférez le garder secret.***	0. Non<br>1. Oui<br>88. Ne sait pas<br>99. Pas de réponse	|____|
**715**	Je ne cherche pas à savoir le résultat, mais avez-vous déjà fait un test de dépistage du VIH ?	0. Non **→ 717**<br>1. Oui **→ 716 et allez à 801**<br>99. Pas de réponse	|____|
**716**	Quand avez-vous subi votre dernier test de dépistage du VIH ?	1. Il y a moins d'un an<br>2. Entre un et deux ans<br>3. Entre deux et quatre ans<br>4. Il y a plus de quatre ans<br>88. Ne sait pas<br>99. Pas de réponse	|____|
**717**	Si vous n'avez jamais fait le test, avez-vous l'intention de faire le test de dépistage du VIH dans les 12 prochains mois ?	0. Non<br>1. Oui<br>88. Ne sait pas<br>99. Pas de réponse	

Section 8 : Stigmatisation et discrimination

**801**	Seriez-vous prête à partager un repas avec une personne que vous savez infectée par le VIH ou malade du SIDA ?	0. Non<br>1. Oui<br>88. Ne sait pas<br>99. Pas de réponse	|____|
**802**	Si un(e) élève ou un(e) étudiant(e) a le VIH, mais n'est pas malade, faut-il l'autoriser à continuer à fréquenter l'école ?	0. Non<br>1. Oui<br>88. Ne sait pas<br>99. Pas de réponse	|____|
**803**	Si une femme de votre famille tombait malade avec le VIH, le virus qui cause le SIDA, seriez-vous prête à vous en occuper dans votre foyer ?	0. Non<br>1. Oui<br>88. Ne sait pas<br>99. Pas de réponse	|____|
**804**	Si un professeur a le VIH, mais n'est pas malade, faut-il l'autoriser à continuer à enseigner à l'école ?	0. Non<br>1. Oui<br>88. Ne sait pas<br>99. Pas de réponse	|____|
**805**	Si vous saviez qu'un commerçant ou un vendeur de produits alimentaires a le VIH, lui achèteriez-vous quand même de la nourriture ?	0. Non<br>1. Oui<br>88. Ne sait pas<br>99. Pas de réponse	|____|
**806**	Si un membre de votre famille tombait malade avec le VIH, préféreriez-vous que la chose reste secrète ?	0. Non<br>1. Oui<br>88. Ne sait pas<br>99. Pas de réponse	|____|

Section 9 : Exposition aux efforts de prévention

901	Avez-vous déjà entendu parler ou vu une publicité sur la prévention du sida ?	0. Non → 903<br>1. Oui<br>99. Pas de réponse → 903	|____|
902	Où avez-vous entendu ou vu cette publicité ?<br>***NE LISEZ PAS LES REPONSES. ENTOUREZ TOUTES LES REPONSES DONNEES.***	a. Radio 0. Non 1. Oui<br>b. Télévision 0. Non 1. Oui<br>c. Journaux 0. Non 1. Oui<br>d. Panneaux de publicité 0. Non 1. Oui<br>e. Affiche/ Brochure 0. Non 1. Oui<br>f. Autres 0. Non 1. Oui<br>Précisez : ________________<br>88. Ne se souvient plus<br>99. Pas de réponse	|____
____
____
____
____
____
____	
903	Avez-vous une fois assisté à une animation de masse sur le sida?	0. Non<br>1. Oui<br>99. Pas de réponse	|____|
904	Avez-vous une fois assisté à une causerie sur le sida?	0. Non → 906<br>1. Oui<br>99. Pas de réponse → 906	|____|
905	Qu'est-ce que vous avez retenu de ces causeries ou animations ?<br>***NE LISEZ PAS LES REPONSES. ENTOUREZ TOUTES LES REPONSES DONNEES.***	a. Modes de transmission du VIH 0. Non 1. Oui<br>b. Moyens de prévention 0. Non 1. Oui<br>c. Le seul moyen de prévention est le préservatif 0. Non 1. Oui<br>d. Une personne apparemment en bonne santé peut être infectée par le VIH 0. Non 1. Oui<br>e. Un bébé peut être infecté par sa mère sans traitement 0. Non 1. Oui<br>f. Autres 0. Non 1. Oui<br>Précisez : ________________<br>88. Ne se souvient plus<br>99. Pas de réponse	|____
____
____
____
____
____
____	
906	Avez-vous déjà entendu ou vu une publicité sur le dépistage volontaire ?	0. Non → 1001<br>1. Oui<br>99. Pas de réponse → 1001	|____|
907	Qu'avez-vous effectivement vu ou entendu dans les publicités sur le dépistage ?	a. Connaitre son statut sérologique 0. Non 1. Oui<br>b. Aller faire le test de dépistage 0. Non 1. Oui<br>c. Le dépistage est gratuit et anonyme 0. Non 1. Oui<br>d. Ne pas avoir peur de faire le test 0. Non 1. Oui<br>e. Autre 0. Non 1. Oui<br>Précisez : ________________<br>88. Ne se souvient plus<br>99. Pas de réponse	|____
____
____
____
____
____	

Section 10 : Conduites addictives

	Consommez-vous de l'alcool	0 Non → **1101**<br>1. Oui	
**1001**	Quelle est la fréquence de votre consommation d'alcool ?	a. Jamais 0<br>b. Une fois par mois 1<br>c. 2 à 4 fois par mois 2<br>d. 2 à 3 fois par semaine 3<br>e. Au moins 4 fois par semaine 4	|____|
**1002**	Combien de verres contenant de l'alcool consommez-vous un jour typique où vous buvez ?	a. 3 ou 4 1<br>b. 5 ou 6 2<br>c. 7 ou 8 3<br>d. 10 ou plus 4	|____|
**1003**	Avec quelle fréquence buvez-vous six verres ou davantage lors d'une occasion particulière ?	a. Jamais 0<br>b. Une fois par mois 1<br>c. Plus d'une fois par mois 2<br>d. Une fois par semaine 3<br>e. Tous les jours ou presque 4	|____|
**1004**	Est-ce que vous avez constaté que vous n'étiez plus capable d'arrêter de boire une fois que vous aviez commencé?	a. Jamais 0<br>b. Une fois par mois 1<br>c. Plus d'une fois par mois 2<br>d. Une fois par semaine 3<br>e. Tous les jours ou presque 4	|____|
**1005**	Au cours de l'année écoulée, combien de fois votre consommation d'alcool vous a-t-elle empêché´ de faire ce qui était normalement attendu de vous ?	a. Jamais 0<br>b. Une fois par mois 1<br>c. Plus d'une fois par mois 2<br>d. Une fois par semaine 3<br>e. Tous les jours ou presque 4	|____|
**1006**	Au cours de l'année écoulée, combien de fois avez-vous eu besoin d'un premier verre pour pouvoir démarrer après avoir beaucoup bu la veille ?	a. Jamais 0<br>b. Une fois par mois 1<br>c. Plus d'une fois par mois 2<br>d. Une fois par semaine 3<br>e. Tous les jours ou presque 4	|____|
**1007**	Au cours de l'année écoulée, combien de fois avez-vous eu un sentiment de culpabilité´ ou des remords après avoir bu ?	a. Jamais 0<br>b. Une fois par mois 1<br>c. Plus d'une fois par mois 2<br>d. Une fois par semaine 3<br>e. Tous les jours ou presque 4<br>f. Non Applicable 5	|____|
**1008**	Au cours de l'année écoulée, combien de fois avez-vous été´ incapable de vous rappeler ce qui s'était passé la soirée précédente parce que vous aviez bu?	a. Jamais 0<br>b. Une fois par mois 1<br>c. Plus d'une fois par mois 2<br>d. Une fois par semaine 3<br>e. Tous les jours ou presque 4	|____|
**1009**	Avez-vous été´ blessé ou quelqu'un d'autre a-t-il été´ blessé parce que vous aviez bu ?	a. Non 0<br>b. Oui, mais pas au cours de l'année écoulée 2<br>c. Oui, au cours de l'année 4	|____|
**1010**		a. Non 0<br>b. Oui, mais pas au cours de l'année écoulée 2	|____|

	Un parent, un ami, un médecin ou un autre soignant s'est-il inquiété de votre consommation d'alcool ou a-t-il suggéré que vous la réduisiez ?	c. Oui, au cours de l'année 4	

Section 11 : Consommation de tabac

**1101**	Fumez-vous **actuellement** du tabac	1. Tous les jours ?<br>2. Moins d'une fois par jour ?<br>3. Pas du tout (non)<br>99. Pas de réponse → **1103 et 1105**	|___|
**1102**	Depuis quand fumez-vous tous les jours	Age de début du tabac |__|__| (ans)<br>Ou<br>Année depuis le début |__|__| années	|___|
**1103**	**Par le passé**, avez-vous fumé du tabac	1. Tous les jours ?<br>2. Moins d'une fois par jours ?<br>3. Pas du tout ?<br>88. Ne sait pas<br>99. Pas de réponse	|___|
**1104**	En moyenne, quelle quantité des produits suivants fumez-vous **actuellement** chaque jour?<br>*NOTEZ OOO **SI AUCUNE** CONSOMMATION*<br>NOTEZ **888** SI CONSOMMATION MAIS PAS TOUS LES JOURS/TOUTES LES SEMAINES.<br>*BIEN VERIFIER QU'IL S'AGIT **D'UNITÉS** DE CIGARETTES ET **PAS DE PAQUETS** DE CIGARETTES.*	a. Cigarettes manufacturées |__|__|__| b. Cigarettes roulées à la main |__|__|__| c. Kreteks |__|__|__| d. Pipes remplies de tabac |__|__|__| e. Cigares, cheeroots, cigarillos |__|__|__| f. Nombre de séances de narguilé (chicha) |__|__|__| g. Autres, préciser :________________ |__|__|__|	|___| |___| |___| |___| |___| |___|
**1105**	A quelle fréquence quelqu'un dans votre entourage fume-t-il à l'intérieur (au travail ou au domicile) ?	1. Tous les jours<br>2. Une fois par semaine<br>3. Une fois par mois<br>4. Moins d'une fois par mois<br>5. Jamais<br>88. Ne sait pas	|___|
**1106**	Au cours des 12 derniers mois, avez-vous essayé d'arrêter de fumer ?	0. Non<br>1. Oui	|___|
**1107**	Au cours des 30 derniers jours, avez-vous remarqué des mises en garde sanitaires sur les paquets de cigarettes ?	0. Non<br>1. Oui<br>3. N'a vu aucun paquet de cigarettes	|___|

Section 12 : Dépression

Connaissance des états de santé mentale durant **les quatre dernières semaines**			
**1201**	Pendant les quatre dernières semaines, vous est-il arrivé de vous **sentir quelquefois épuisé sans raison valable** ?	0. Jamais<br>1. Rarement<br>2. Parfois<br>3. La plupart du temps<br>4. Tout le temps	|____|
**1202**	Pendant les quatre dernières semaines, vous est-il arrivé de vous **sentir nerveux** ?	0. Jamais<br>1. Rarement<br>2. Parfois<br>3. La plupart du temps<br>4. Tout le temps	|____|
**1203**	Pendant les quatre dernières semaines, vous est-il arrivé de **ressentir une nervosité si grande que rien ne pouvait vous calmer ?**	0. Jamais<br>1. Rarement<br>2. Parfois<br>3. La plupart du temps<br>4. Tout le temps	|____|
**1204**	Pendant les quatre dernières semaines, vous est-il arrivé de de vous **sentir quelquefois désespéré** ?	0. Jamais<br>1. Rarement<br>2. Parfois<br>3. La plupart du temps<br>4. Tout le temps	|____|
**1205**	Pendant les quatre dernières semaines, vous est-il arrivé de vous **sentir des fois agité** ?	0. Jamais<br>1. Rarement<br>2. Parfois<br>3. La plupart du temps<br>4. Tout le temps	|____|
**1206**	Pendant les quatre dernières semaines, vous est-il arrivé de ressentir une **agitation si grande que vous ne pouviez pas vous maintenir en place**?	0. Jamais<br>1. Rarement<br>2. Parfois<br>3. La plupart du temps<br>4. Tout le temps	|____|
**1207**	Pendant les quatre dernières semaines, vous est-il arrivé **de vous sentir parfois déprimé** ?	0. Jamais<br>1. Rarement<br>2. Parfois<br>3. La plupart du temps<br>4. Tout le temps	|____|
**1208**	Pendant les quatre dernières semaines, vous est-il arrivé d'avoir **l'impression que vous vous efforciez à tout** ?	0. Jamais<br>1. Rarement<br>2. Parfois<br>3. La plupart du temps<br>4. Tout le temps	|____|
**1209**	Pendant les quatre dernières semaines, vous est-il arrivé **de ressentir une tristesse si grande que rien ne pouvait vous remonter le moral ?**	0. Jamais<br>1. Rarement<br>2. Parfois<br>3. La plupart du temps<br>4. Tout le temps	|____|
**1209**	Pendant les quatre dernières semaines, vous est-il arrivé de vous **sentir bon à rien** ?	0. Jamais<br>1. Rarement<br>2. Parfois<br>3. La plupart du temps<br>4. Tout le temps	|____|

La manière dont vous vous êtes senti pendant les **quatre dernières semaines**.			
Si vous avez répondu « **Jamais** » **aux dix questions précédentes**, vous n'avez pas besoin de répondre à ces questions supplémentaires.			
**1211**	Pendant les quatre dernières semaines, combien de jours avez-vous été TOTALEMENT INCAPABLE de travailler, d'étudier ou de vaquer à vos activités quotidiennes à cause de ces malaises ?	|__________| (Nombre de jour)	
**1212**	[En dehors de ces jours-là], pendant les 4 dernières semaines, COMBIEN DE JOURS avez-vous pu travailler, étudier ou vaquer à vos activités quotidiennes en RÉDUISANT ces activités à cause de ces malaises ?	|__________| (Nombre de jour)	
**1213**	Pendant les 4 dernières semaines, combien de fois avez-vous consulté un médecin ou un autre professionnel de la santé concernant ces malaises ?	|__________| (Nombre de consultation)	
**1214**	Pendant les 4 dernières semaines, vous est-il arrivé que des problèmes de santé physiques étaient la cause principale de ces malaises ?	0. Jamais<br>1. Rarement<br>2. Parfois<br>3. La plupart du temps<br>4. Tout le temps	|____|

Section 13: Accès aux soins

**1301**	Quand êtes-vous allé dans une structure de santé pour la dernière fois ?	0. < une semaine<br>1. Entre une semaine et un mois<br>2. Entre un mois et 3 mois<br>3. Entre 3 mois et 6 mois<br>4. > 6 mois<br>88. Ne se souvient pas<br>99. Pas de réponse	|____|
**1302**	Où consultez-vous quand vous avez un problème de santé ?	0. CHU ou CHR<br>1. Dans une clinique privée dans mon quartier<br>2. Dans une clinique privée en dehors du quartier<br>3. Dans un centre médico-social dans mon quartier<br>4. Dans un centre médico-social en dehors de mon quartier<br>5. Je ne consulte pas et j'achète les médicaments au marché/pharmacie<br>6. Autre, préciser :______________________________________<br>99. Pas de réponse	|____|
**1303**	Avez-vous ou connaissez-vous un professionnel de santé que vous consultez quand vous avez un problème de santé ?	0. Non<br>1. Oui, un médecin<br>2. Oui un infirmier<br>3. Oui, je ne connais pas son titre	|____|

**1304**	Avez-vous déclaré à votre médecin traitant ou au professionnel de santé que vous êtes PS?	0. Non<br>1. Oui<br>88. Ne se souvient pas<br>99. Pas de réponse	|____|
**1305**	Pourquoi ne leur avez-vous pas déclaré que vous êtes PS?	____________________<br>____________________	
**1306**	Vous a-t-il déjà proposé de faire les tests de dépistage des IST	a. Syphilis 0. Non 1. Oui<br>b. Chlamydiae 0. Non 1. Oui<br>c. Gonococcie 0. Non 1. Oui<br>d. Hépatite B 0. Non 1. Oui<br>e. Condylome 0. Non 1. Oui<br>f. Hépatite C 0. Non 1. Oui<br>g. VIH 0. Non 1. Oui<br>h. Autre 0. Non 1. Oui<br>Précisez : ____________________<br>99. Pas de réponse 0. Non 1. Oui	|____| |____| |____| |____| |____| |____| |____| <br>|____|

Section 14 : Tests VIH et soins

**1401**	Connaissez-vous un endroit où vous pourriez faire le test de dépistage du VIH ?	0. Non<br>1. Oui	|____|
**1402**	Avez-vous déjà effectué un test de dépistage du VIH?<br>*Ne poser aucune question sur les résultats du test.*	0. Non → **1406**<br>1. Oui<br>99. Ne souhaite pas répondre → **1406**	|____|
**1403**	À quelle occasion avez-vous effectué votre dernier test de dépistage ?<br>*Une seule réponse possible.*	1. À l'occasion d'un don de sang<br>2. Après un rapport non protégé<br>3. Lors d'une campagne de dépistage gratuit et anonyme<br>4. Décision volontaire<br>5. Lors d'un suivi médical<br>6. Sur proposition (après sensibilisation) d'un personnel médical<br>7. Suite à une action de sensibilisation<br>8. Autre<br>Précisez :	|____|
**1404**	À quand remonte votre dernier test ?<br>*Si refus de répondre, entourer 99.*	1. Moins de 3 mois<br>2. Entre 3 et 6 mois<br>3. Entre 6 et 12 mois<br>4. Plus d'un an<br>96. Ne s'en souvient plus<br>99. Ne souhaite pas répondre	|____|
**1405**	Êtes-vous retourné chercher les résultats de votre test ?<br>*Ne poser aucune question sur les résultats du test.*	0. Non<br>1. Oui<br>99. Ne souhaite pas répondre	|____|
**1406**	Connaissez-vous l'existence d'un traitement destiné aux personnes qui ont le virus du sida (VIH) ?	0. Non<br>1. Oui	|____|
**1407**	Connaissez-vous le statut sérologique de votre partenaire régulier ?<br>*Si plusieurs partenaires, poser la question pour le partenaire principal.*	0. Non<br>1. Oui	|____|
**1408**	Accepteriez-vous de faire le test de dépistage du VIH	0. Non<br>1. Oui	|____|
**1409**	Si oui, souhaiteriez-vous connaitre le résultat de votre test de dépistage du VIH ?	0. Non<br>1. Oui	|____|
**1410**	Si non, pourquoi ?	____________________<br>____________________	

NB :

1- Vérifier que toutes les questions sont bien remplies

2- Remercier l'enquêté

3- Inviter l'enquêté à s'orienter vers la salle de prélèvement en lui remettant un coupon
