# Supplementary material for: Prevalence of hepatitis B and C among female sex workers in Togo, West Africa
Source: PLoS One. 2021 Dec 10;16(12):e0259891. doi: 10.1371/journal.pone.0259891 (PMC8664183; doi:10.1371/journal.pone.0259891)
Supplement: S2 File — (PDF) [file pone.0259891.s002.pdf]

QUESTIONNAIRE FEMALE SEX WORKERS, Togo 2017

ENGLISH TRANSLATION

City name: ___________________________________

identifier: ___________________________________

Date of interview/data collection: ____ / ____ / 2017

investigator: ___________________ [code: ....]

Section 1: Socio-demographic characteristics

No	Questions and references	Coded responses	jump
**101**	How old are you?<br><br>***End of questionnaire if age less than 18 years or born after August 1999.***	|___|___|/|___|___|/|___|___|___|___| Day Month Year<br>**|__|__|** Age in years<br>88. Don't know<br>99. No answer	|____|
**102**	Have you ever been to school?	0. No<br>1. Yes<br>99. No answer	**104**<br>|____|
**103**	What level of education have you achieved?<br>***CIRCLE A SINGLE ANSWER.***	1. Primary<br>2. Secondary	|____|

		3. University/College<br>99. No Response	
**104**	What is your religion?<br><br>***CIRCLE A SINGLE ANSWER.***	0. Non believer<br>1. Animist<br>2. Christian<br>4. Muslim<br>5. Other, specify: ___________________________________	|____|
**105**	What is your nationality?	1.Togolese<br>2. Other, specify: ____________________________________	|____|
**106**	What is your ethnicity?<br><br>***CIRCLE A SINGLE ANSWER.***	1. Adja-Ewe<br>2. Akposso<br>3. Ana-Ife<br>4. Kabyè<br>5. Kotokoli<br>6. Mina<br>7. Moba<br>6. Tchamba<br>8. Other, Précisez : ___________________________________	|____|

**107**	Have you ever been married?	0. No<br>1. Yes<br>99. No answer	|____|
**108**	What is your current marital status?	1. Married and living with spouse<br>2. Married and living with another sexual partner<br>3. Married and not living with spouse or with another sexual partner<br>4. Unmarried and living with a sexual partner<br>5. Unmarried and not living with a partner sexual<br>99. No response	Ú**110**
**109**	Does your husband (or your partner) have more than one wife/partner ?	0. No<br>1.Yes<br>88. Don't know<br>99. No response	|____|
**110**	How many children do you have in total?<br>(your own children)	|__|__| children<br>***MARK 00 IF NO CHILDREN.***	

Section 1: : Sociodemographic characteristics (continued)

**111**	At what age did you start offering paid sex?	|__|__| Age in years<br>88. Do not know<br>99. No reply	|____|
**112**	Do you have any additional activities bringing you additional income?	0. No<br>1. Yes<br>99. No reply	|____|
**113**	What are these activities?<br><br>***MULTIPLE POSSIBLE ANSWERS.***	1. Executive in a private structure 0. No 1. Yes<br>2. Executive in a public structure 0. No 1. Yes<br>3. Trade 0. No 1. Yes<br>4. Services (hairdresser, seamstress...) 0. No 1. Yes<br>5. Unskilled manual work 0. No 1. Yes<br>6. Skilled manual work 0. No 1. Yes<br>7. House help 0. No 1. Yes<br>8. Agricultural work 0. No 1. Yes<br>9. Other, 0. No 1. Yes<br>specify: ____	|____| |____| |____| |____| |____| |____| |____| |____| |____|

**114**	Are you the financial support of your family (children, parents) or the financial support of others?	0. No<br>1. Yes<br>99. No reply	|____|
**115**	How many people currently depend on your income?	|__|__| persons<br>88. Don't know<br>99. No reply	|____|

Section 2: Sexual History; number and types of partners

**I would now like to** ask you a few questions about your sexual **partners.**			
**201**	At what age did you have your first sexual intercourse?	|__|__| Age in years<br>88. Do not know<br>99. No reply	|____|
**202**	Among all of your sexual partners of these last 7 days, how many were...<br>- CLIENTS (partners with whom you have a sexual relation in exchange for money)?<br>- other PARTNERS (those with whom you had sexual intercourse, but not for in exchange of money)?<br>***INCLUDE*** IN THE LATTER THE SPOUSE ***OR COMMON-LAW PARTNER(S).***	**|__|__| Customers**<br>88. Don't know<br>99. No reply<br>**|__|__| OTHER PARTNERS**<br>88. Do not know<br>99. No reply	|____| |____|
**203**	In total, how many different sexual partners have you had in the last seven (7) days?<br>***INCLUDE SPOUSES AND COMMON-LAW PARTNER. VERIFY THAT THE NUMBERS*** TO ***TTO OBTAINED AVEC THE Q202 AND AVEC THE Q203 ARE EQUAL.***	**|__|__| TOTAL NUMBER OF PARTNERS**<br>88. Don't know<br>99. No reply	|____|

Section 4: Sexual intercourse with other partners

	**REFERENCE: SEE Question 202**<br><br>TO A SEXUAL CLIENT(S) OTHER THAN HIS OR HER CLIENTS |__|	HAS NO SEXUAL RA R TENAIRE OTHER THAN ITS CLIENTS<br><br>|__| Ú **501**	
**401**	Try to remember the last person with whom you had non-paying sex. How many relationships have you had with this person inthe last thirty (30) days ?	|__|__| Number of times<br>88. Do not know<br>99. No reply	|____|

Section 3: Paid sex

**301**	How many customers did you have during your last day of work?	|__|__| **NUMBER OF CUSTOMERS**<br>88. Do not know<br>99. No reply	|____|
**302**	How much have you earned with your last customer?	|__|__|__|__|__|__|__| Francs CFA<br>88. Do not know	|____|

		99. No reply	

Section 5: Condoms

**Male condoms: Knowledge and availability**			
**501**	Have you ever heard of the male condom?<br><br>***SHOW SPECIMEN OR PHOTO.***	0. No<br>1. Yes<br>88. Do not know<br>99. No reply	|____|
**502**	Have you ever used a male condom with **any** of your sexual partners?	0. No<br>1. Yes<br>88. Do not know<br>99. No reply	|____|
**503**	Do you know of a place or a person who can give you male condoms?<br><br>***CIRCLE A SINGLE ANSWER.***	0. No<br>1. Yes<br>99. No reply	|____|
**504**			

	In which places or from whom can you get male condoms?<br><br>***REPEAT THE QUESTION AND MARK ALL THE ANSWERS GIVEN.***	a. shop 0. No 1. Yes<br>B. pharmacy 0. No 1. Yes<br>c. Market 0. No 1. Yes<br>d. Hospital 0. No 1. Yes<br>E. dispensary 0. No 1. Yes<br>f. Family Planning Centre 0. No 1. Yes<br>g. Bar/ Hotel 0. No 1. Yes<br>H. Young educator 0. No 1. Yes<br>i. Friend 0. No 1. Yes<br>I. other 0. No 1. Yes<br>Specify: ____<br>99. No answer 0. No 1. Yes	|____| |____| |____| |____| |____| |____| |____| |____| |____| |____|
**505**	How long does it take you to get a male condom from your home or workplace?	1. Less than an hour<br>2. Between an hour and a day<br>3. More than a day<br>88. Do not know<br>99. No reply	|____|
**506**	How do you get male condoms?	1. Per purchase	

		2. Free of charge<br>3. By purchase and free of charge	|____|
**507**	How much does it cost you to buy male condoms per month?		
**Level of confidence in condom protection**			
		97. Not applicable<br>88. Do not know	
**508**	How many male condoms do you have in your room and/or with you?	|__|__|__| availablemeadowse rvatifs<br>99. No answer	|____|

**509**	According to you, condom use protects against the occurrence of HIV infection	1. Yes between 80% and 100%<br>2. Yes but between 60% and 80%<br>3. Yes to 50%<br>4. No, does not protect<br>99. Don't know	**|___|**
**Condom and type of sexual intercourse**			
**510**	What kind of sexual intercourse have you had?<br>*SEVERAL POSSIBLE ANSWERS.*	1. Oral<br>2. Vaginal<br>3. Anal	**|___|**

**511**	Did you use a condom for this oral sex (fellatio, anulingus, cunilingus)?	1. Systematically<br>2. Often<br>3. Occasionally<br>4. Never<br>5. Not applicable	
**512**	Did you use a condom for this anal sex?	1. Systematically<br>2. Often<br>3. Occasionally<br>4. Never<br>5. Not applicable	|___|
**513**	Do you use a condom for vaginal intercourse?	1. Systematically<br>2. Often<br>3. Occasionally<br>4. Never	|___|
**514**	Who was this partner?	1. A known partner<br>2. A new partner<br>3. A stranger<br>4. A client	

Condom and last sexual intercourse

**513**	Did you use a condom during the last sexual intercourse?	0. No Ú **514**<br>1. Yes<br>88. Do not know **Ú 515**<br>99. No reply	|___|
**514**	Why didn't you use a condom?	1. Refusal of the partner<br>2. Condom not available<br>3. I know the status of the partner<br>4. Other, specify: ___________________________	|___|
**515**			
**516**			
**Condom and last sexual intercourse with a client**			
**517**	When you hadsexwith a client, did you use a condom?	0. No Ú **519**<br>1. Yes<br>88. Ne<br>sait pas Ú **518** Ú **306**<br>99. No reply	|_____|
**518**	Who proposed the use of a condom during this report?	1. Myself<br>2. Partenaire<br>3. Joint Decision Ú **520**	

	CIRCLE A SINGLE ANSWER.	88. Do not know<br>99. No reply	|____|
**519**	Why did you not use a condom for this report?<br><br>***CIRCLE ALL THE ANSWERS GIVEN.***	a. No condom available0. No 1. Yes<br>B. Condom too expensive 0. No 1. Yes<br>c. Partner Objection 0. No 1. Yes<br>d. Does not like condom 0. No 1. Yes<br>E. Used another contracept0. No 1. Yes<br>f. Deemed condom useless 0. No 1. Yes<br>g. Don't think about it 0. No 1. Yes<br>h. Other 0. No 1. Yes<br>Specify:______________________________88. Don't know<br>99. No answer	|____| |____| |____| _<br>|____| |____| |____| |____| |____| |____|
**520**	During the last thirty (30) days, have you frequently used a condom with your **clients?** ?	1. Each time<br>2. Often<br>3. Rarely<br>4. Never<br>88. Do not know	|____|

		99. No reply	
**Condoms and sex with other partners**			
**521**	When you had sex with another partner, did you use a condom?	0. No<br>1. Yes<br>88. Do not know<br>99. No reply	|____|
**522**	Who suggested the use of a condom during this report?<br><br>***CIRCLE A SINGLE ANSWER.***	1. Myself<br>2. Partner<br>3. Joint Decision<br>88. Do not know<br>99. No reply	|____|
**523**	Why did you not use a condom for this intercourse?<br><br>***CIRCLE ALL THE ANSWERS GIVEN.***	a. No condom available 0. No 1. Yes<br>B. Condom too expensive 0. No 1. Yes<br>c. Partner Objection 0. No 1. Yes<br>d. Does not like condom 0. No 1. Yes<br>E. Used another contraception 0. No 1. Yes<br>f. Deemed condom useless 0. No 1. Yes<br>g. Did not think about it 0. No 1. Yes	|____| |____| |____| _<br>|____| |____| |____|

		h. Other 0. No 1. Yes<br>Specify:________________________________88. Don't know<br>99. No answer	|____| |____| |____|
**524**	During the last thirty (30) days, how often have you used a condom with your other partners?	1. Each time<br>2. Often<br>3. Rarely<br>4. Never<br>88. Do not know<br>99. No reply	|____|
**Last sexual intercourse without a condom**			
**525**	When was the last time you had sex without using a condom?	0. Last Night<br>1. Less than a week<br>2. Less than a month<br>3. Less than six months<br>4. I don't remember	**|___|**
**526**	Why didn't you use a condom?	1. Refusal of the partner<br>2. Condom not available	**|___|**

		3. I know the status of the partner<br>4. Other,<br>specify: ___________________________	
**527**	What kind of sexual intercourse have you had?	1. Oral<br>2. Vaginal<br>3. Anal	**I___I**
**528**	Who was this partner?<br>*SEVERAL POSSIBLE ANSWERS.*	1. A known partner<br>2. A new partner<br>3. A stranger<br>4. A client	**I___I**

**Circumstances of sex without a condom**			
**529**	Under what circumstances would you agree to have sex without a condom?	0. Never<br>1. Forced intercourse<br>2. For money<br>3. With a partner I know well<br>4. I know the PARTNER's HIV status<br>5. Other,<br>specify: ______________________________	**I___I**

**Condom accident**			
**530**	Do the condoms you use ever tear during sex?	0. No, never<br>1. Yes, rarely<br>2. Yes, often<br>3. Yes, very frequently	**|___|**
**Female condoms**			
**531**	Have you ever heard of the female condom ?<br><br>***SHOW A SPECIMEN*** OR ***PHOTO.***	0. No<br>1. Yes<br>88. Do not know<br>99. No reply	|____|
**532**	Have you ever used the female condom?<br><br>***SHOW A SPECIMEN*** OR ***PHOTO.***	0. No<br>1. Yes<br>2. Not applicable<br>88. Don't know<br>99. No answer	|____|
**533**	Do you know of a place or a person who can give you a female condom?	0. No<br>1. Yes<br>2. Not applicable	|____|

		99. No answer		
**534**	In which places or from whom can you get female condoms?<br><br>***REPEAT THE QUESTION AND MARK ALL THE ANSWERS GIVEN .***	a. shop	0. No 1. Yes	|____|
		B. pharmacy	0. No 1. Yes	|____|
		c. Market	0. No 1. Yes	|____|
		d. Hospital	0. No 1. Yes	|____|
		E. dispensary	0. No 1. Yes	|____|
		f. Family Planning Centre	0. No 1. Yes	|____|
		g. Bar / Hotel	0. No 1. Yes	|____|
		H. Young educator	0. No 1. Yes	|____|
		i. Friend	0. No 1. Yes	|___|
		H. other	0. No 1. Yes	|____|
		Specify:_________________________________		
		i. Not applicable	0. No 1. Yes	|____|
		99. No answer	0. No 1. Yes	|____|

Section 6: IST

**601**	Have you ever heard of diseases that can be transmitted during sexual intercourse called sexually transmitted infections (STIs)?	0. Non<br>1. Oui<br>99. No reply	|____|
**602**	Do you know of any symptoms of STIs in women?<br>Do you know of any others?<br>***DON'T READ THE SYMPTOMS.***<br>***CIRCLE (1) FOR EACH SYMPTOM MENTIONED.***<br>***CIRCLESZ (0) FORR ANY SYMPTOME NON MENTIONS.***<br>***ANSWERS MULTIPLES POSSIBLE.***	a. Abdominal pain 0. No 1. Yes<br>B. Smelly losses 0. No 1. Yes<br>c. Vaginal discharge 0. No 1. Yes<br>d. Itching 0. No 1. Yes<br>E. Painful urination 0. No 1. Yes<br>f. Genital lesions 0. No 1. Yes<br>g. Swelling in the groin 0. No 1. Yes<br>H. Other 0. No 1. Yes<br>Specify: ____<br>99. No answer 0. No 1. Yes	|____| |____| |____| |____| |____| |____| |____| |____| |____|
**603**	Do you know of any symptoms of STIs in humans?<br>Do you know of any others?<br>***DON'T READ PAS THE SYMPTOMS.***	a. Urethral flow 0. No 1. Yes<br>B. Swelling in the groin 0. No 1. Yes<br>c. Painful urination 0. No 1. Yes	|____| |____| |____|

	CIRCLE (1) FOR EACH SYMPTOM MENTIONED.<br><br>***CIRCLESZ (0) FORR ANY SYMPTOME NON MENTIONS.***<br><br>***ANSWERS MULTIPLES POSSIBLE.***	d. Itching 0. No 1. Yes<br>E. other 0. No 1. Yes<br>Specify:<br>____<br>99. No answer 0. No 1. Yes	|____| |____| |____|
**604**	Have you had vaginal **discharge** during the last twelve (12) months ?	0. No<br>1. Yes<br>88. Do not know<br>99. No reply	|____|
**605**	Have you had **ulcerations/lesions** in the genital area during the last twelve (12) months?	0. No<br>1. Yes<br>88. Do not know<br>99. No reply	|____|
**606**	The last time you had loss/discharge or genital ulceration/lesion, did you?<br><br>a. Consult in a hospital or health center to obtain advice and/or medicines?	No Yes Don't know No answer<br>0 1 88 99<br>0 1 88 99<br>0 1 88 99<br>0 1 88 99	|____|

	b. Consult a student to obtain advice and/or medicines?	0<br>0	1<br>1	88<br>88	99<br>99	|____|
	c. consult un a traditional<br>healer to obtain advice and/or medicines?	0	1	88	99	|____|
	d. Took some medicines that you already had in your possession?					|____|
	e. discussed with your sexual partner of this discharge or the lesion (IST)?					|____|
	f. Stopped any sexual relations as long as the symptoms remained? that the symptom s existeaien t ?					|____|
	j. used as a reservative during your sexual intercourse as long as the symptoms have persisted?					|____|
	READ THE FOLLOWINGVANTS. POSSIBLE ANSWERS.					

**607**	What was your first step?<br><br>***ONLY ONE ANSWER POSSIBLE.***	1. Consultation in a health center<br>to obtain advice and/or medicines?<br>2. Consultation in a pharmacy<br>to get advice and/or medicines?<br>3. Consultation to a traditional healer<br>to obtain advice and/or medicines?<br>4. Took medicine already in your possession ?<br>5. Other, specify: __________________<br>88. Does not remember<br>99. No reply	|____|
**608**	If you took medication the last time the symptoms appeared, who gave them to you?<br><br>***CIRCLE ALL THE ANSWERS GIVEN.***	a. dispensary 0. No 1. Yes<br>B. pharmacy 0. No 1. Yes<br>c. Traditional Healer 0. No 1. Yes<br>d. Friend or relative 0. No 1. Yes<br>E. Medicine at home 0. No 1. Yes<br>f. Did not take medication 0. No 1. Yes<br>88. Does not remember 0. No 1. Yes<br>99. No answer 0. No 1. Yes	|____| |____| |____| |____| |____| |____| |____|
**609**	How much did you pay for your medications?		

		|__|__|__| __|__|__| Francs CFA	
**610**	**REFERENCE: SEE QUESTIONS 606, 607, 608**<br>Consulted a health worker at a clinic or hospital	0. No<br>1. Yes	|____|
**611**	How long did you wait between the first onset of symptoms and your visit to a clinic or hospital?	1. 1 week or less<br>2. Between a week and a month<br>3. 1 month or more<br>88. Don't know<br>99. No answer	|____|
**612**	Has a prescription been issued for your medications?	0. No<br>1. Yes<br>88. Do not know<br>99. No answer	|____|
**613**	Were you able to get the prescribed medications?	0. No<br>1. Yes<br>3. Partially<br>88. Does not remember<br>99. No answer	|____|
**614**			

	If you did not get all the prescribed medication, what were the reasons?<br><br>***ENTOUREZ TOUTES LES REPONSES DONNEES.***	a. No money to buy them 0. No 1.Yes<br>b. Medicine not available 0. No 1. Yes<br>c. Does not like to take medicine 0. No 1. Yes<br>d. Other 0. No 1. Yes<br>Specify : ____________________________<br>88. Does not remember<br>99. No answer	|____| |____| |____| |____| |____|

Section 7: Knowledge and attitude on HIV and AIDS

**701**	Have you ever heard of a disease called AIDS?	0. No.<br>1. Yes<br>99. No answer	|____|
**702**	Do you know someone who is HIV positive or who died from AIDS ?	0. No.<br>1. Yes<br>88. Does not know<br>99. No answer	|____|

**703**	Do you have a relative or close friend who is infected with HIV or who has died of AIDS?	0. No<br>1. Yes<br>88. Don't know<br>99. No answer	|_____|
**704**	Can we protect ourselves against HIV, the virus that causes AIDS, by using a condom correctly and systematically (with every sexual intercourse)?	0. No<br>1. Yes<br>88. Don't know<br>99. No answer	|_____|
**705**	Can one be contaminated with the<br>HIV through a mosquito bite?	0. No<br>1. Yes<br>88. Don't know<br>99. No answer	|_____|
**706**	Can we protect ourselves against HIV by having sex exclusively with someone who is both faithful and uninfected?	0. No<br>1. Yes<br>88. Don't know<br>99. No answer	|_____|
**707**	Can we protect ourselves against HIV by abstaining from sexual intercourse?	0. No<br>1. Yes<br>88. Don't know<br>99. No answer	|_____|

**708**	Can I become infected with HIV by sharing a meal with an infected person?	0. No<br>1. Yes<br>88. Don't know<br>99. No answer	|_____|
**709**	Can you become infected with HIV by sharing a hypodermic needle already used by someone else?	0. No<br>1. Yes<br>88. Don't know<br>99. No answer	|_____|
**710**	Do you think that a person who appears to be healthy can actually be infected with HIV, the virus that causes AIDS?	0. No<br>1. Yes<br>88. Don't know<br>99. No answer	|_____|
**711**	If a pregnant woman has HIV or AIDS, can she transmit the virus to the child she is carrying?	0. No Ú **713**<br>1. Yes<br>88. Don't know<br>99. No answer Ú **713**	|_____|
**712**	What can a pregnant woman do to reduce the risk of HIV transmission to the child she is carrying?<br>***DO NOT READ THE ANSWERS. CIRCLE ALL THE*** ANSWERS ***GIVEN.***	a. Antiretroviral treatment 0. No 1. Yes<br>B. Other 0. No 1. Yes<br>Specify:<br>____	|_____
_____	

		88. Don't know<br>99. No answer	|____|
**713**	If a woman has HIV or AIDS, can she transmit the virus to her baby when she breastfeeds?	0. No<br>1. Yes<br>88. Don't know<br>99. No answer	|____|
**714**	Is it possible in your community to have a confidential test to find out if you are infected with HIV?<br>***By confidential, I mean that no one can know the result of the test if you prefer to keep it secret.***	0. No<br>1. Yes<br>88. Don't know<br>99. No answer	|____|
**715**	I'm not trying to find out the result, but have you ever been tested for HIV?	0. No **Ú 717**<br>1. Yes Ú **716 and go to 801**<br>99. No answer	|____|
**716**	When was your last HIV test?	1. Less than a year ago<br>2. Between one and two years<br>3. Between two and four years<br>4. More than four years ago<br>88. Don't know	|____|

		99. No answer	
**717**	If you have never been tested, do you intend to be tested for HIV in the next 12 months?	0. No<br>1. Yes<br>88. Don't know<br>99. No answer	

Section 8: Stigma and Discrimination

**801**	Would you be willing to share a meal with someone you know is infected with HIV or has AIDS?	0. No<br>1. Yes<br>88. Don't know<br>99. No answer	|_____|
**802**	If a student has HIV but is not ill, should they be allowed to continue attending school?	0. No	

		1. Yes<br>88. Don't know<br>99. No answer	|____|
**803**	If a woman in your family were to become ill with HIV, the virus that causes AIDS, would you be willing to take care of it in your home?	0. No<br>1. Yes<br>88. Don't know<br>99. No answer	|____|
**804**	If a teacher has HIV but is not sick, should he or she be allowed to continue teaching at school?	0. No<br>1. Yes<br>88. Don't know<br>99. No answer	|____|
**805**	If you knew that a food shopkeeper or seller has HIV, would you still buy them food?	0. No<br>1. Yes<br>88. Don't know<br>99. No answer	|____|
**806**	If a family member became ill with HIV, would you prefer to keep it secret?	0. No<br>1. Yes<br>88. Don't know<br>99. No answer	|____|

Section 9: Exposure to Prevention Efforts

**901**	Have you ever heard of or seen an advertisement about AIDS prevention?	0. No **Ú 903**<br>1. Yes<br>99. No answer Ú **903**		|____|
**902**	Where have you heard or seen this ad?<br><br>***DO NOT READ THE ANSWERS. CIRCLE ALL THE*** ANSWERS ***GIVEN.***	a. radio	0. No 1. Yes	|____|
		B. television	0. No 1. Yes	|____|
		c. Newspapers	0. No 1. Yes	|____|
		d. Billboards	0. No 1. Yes	|____|
		e. Poster/ Brochure	0. No 1. Yes	|____|
		f. Other	0. No 1. Yes	|____|
		Specify: ____		
		88. Can't remember		|____|
		99. No answer		
**903**	Have you once attended a mass animation on AIDS?	0. No<br>1. Yes<br>99. No answer		|____|

**904**	Have you once attended an AIDS talk?	0. No Ú **906**<br>1. Yes<br>99. No answer Ú**906**	|____|
**905**	What did you learn from these talks or animations?<br><br>***DO NOT READ THE ANSWERS. CIRCLE ALL THE*** ANSWERS ***GIVEN.***	a. Modes of HIV transmission 0. No 1. Yes<br>B. Means of prevention 0. No 1. Yes<br>c. The only means of prevention is condoms 0. No 1. Yes<br>d. An apparently healthy person may be infected with HIV 0. No 1. Yes<br>E. A baby can be infected by his mother without treatment 0. No 1. Yes<br>f. Other 0. No 1. Yes<br>Specify: ____<br>88. Can't remember<br>99. No answer	|____| |____| |____| |____| |____| |____| |____|
**906**	Have you ever heard or seen an advertisement about voluntary testing?	0. No Ú **1001**<br>1. Yes<br>99. No answer Ú **1001**	|____|

**907**	What did you actually see or hear in the screening ads?	a. Know your HIV status 0. No 1. Yes<br>B. Go for the screening test 0. No 1. Yes<br>c. Screening is free and anonymous 0. No 1. Yes<br>d. Don't be afraid to take the test 0. No 1. Yes<br>E. other 0. No 1. Yes<br>Specify: ____<br>88. Can't remember<br>99. No answer	|____| |____| |____| |____| |____| |____|

Section 10: Addictive Behaviours

	Consume alcohol yourself	0 No Ú **1101**<br>1. Yes	
**1001**	How often do you drink alcohol?	a. never 0<br>B. Once a month 1	

		c. 2 to 4 times a month	2	|___|
		d. 2 to 3 times a week	3	
		E. At least 4 times a week	4	
**1002**	How many glasses containing alcohol do you consume on a typical day when you drink?	s. 3 or 4	1	
		b. 5 or 6	2	|___|
		c. 7 or 8	3	
		d. 10 or more	4	
**1003**	How often do you drink six or more drinks on a particular occasion?	a. never	0	
		B. Once a month	1	
		c. More than once a month	2	|___|
		d. Once a week	3	
		E. Almost every day	4	
**1004**	Did you find that you were no longer able to stop drinking once you started?	a. never	0	
		B. Once a month	1	
		c. More than once a month	2	|___|
		d. Once a week	3	
		E. Almost every day	4	

**1005**	Over the past year, how many times has your alcohol consumption prevented you from doing what was normally expected of you?	a. never<br>B. Once a month<br>c. More than once a month<br>d. Once a week<br>E. Almost every day	0<br>1<br>2<br>3<br>4	|___|
**1006**	Over the past year, how many times have you needed a first drink to be able to start after drinking a lot the day before?	a. never<br>B. Once a month<br>c. More than once a month<br>d. Once a week<br>E. Almost every day	0<br>1<br>2<br>3<br>4	|___|
**1007**	How many times in the past year have you felt guilty or remorseful after drinking?	a. never<br>B. Once a month<br>c. More than once a month<br>d. Once a week<br>E. Almost every day<br>f. not applicable	0<br>1<br>2<br>3<br>4<br>5	|___|
**1008**				

	How many times in the past year have you been unable to remember what happened the previous evening because you had been drinking?	a. never<br>B. Once a month<br>c. More than once a month<br>d. Once a week<br>E. Almost every day	0<br>1<br>2<br>3<br>4	|___|
**1009**	Were you injured or was someone else injured because you had been drinking?	a. No<br>B. Yes, but not in the past year<br>c. Yes, during the year	0<br>2<br>4	|___|
**1010**	Has any relative, friend, doctor or other caregiver been concerned about your alcohol consumption or suggested that you reduce it?	a. No<br>B. Yes, but not in the past year<br>c. Yes, during the year	0<br>2<br>4	|___|

Section 11: Tobacco Consumption

**1101**	Do you **currently smoke** tobacco	1. Every day?<br>2. Less than once a day?<br>3. Not at all (no)<br>99. No answer Ú **1103 and 1105**	|_____|

**1102**	How long have you been smoking every day	Age of smoking|__|__| (years)<br>or<br>Year since the beginning |__|__| years	|____|
**1103**	**In the past,** have you smoked tobacco	1. Every day?<br>2. Less than once a day?<br>3. Not at all?<br>88. Don't know<br>99. No answer	|____|
**1104**	On average, how many of the following products do you **currently** smoke each day?<br>*NOTE 000 **IF NO** CONSUMPTION*<br>NOTE **888** IF CONSUMPTION BUT NOT EVERY DAY/WEEKLY**.**<br>*MAKE SURE THAT THESE ARE **UNITS OF** CIGARETTES AND NOT PACKS **OF** CIGARETTES.*	a. Manufactured cigarettes |__|__|__| b. Hand-rolled cigarettes |__|__|__| c. Kreteks |__|__|__| d. Pipes filled with tobacco |__|__|__| E. Cigars, cheeroots, cigarillos |__|__|__| f. Number of sessions of hookah (shisha) |__|__|__| g. Other, specify:____________________ |__|__|__|	|____| |____| |____| |____| |____| |____|
**1105**	How often does someone around you smoke indoors (at work or at home)?	1. Every day<br>2. Once a week<br>3. Once a month	|____|

		4. Less than once a month<br>5. Never<br>88. Don't know	
**1106**	In the last 12 months, have you tried to quit smoking?	0. No<br>1. Yes	|____|
**1107**	In the last 30 days, have you noticed any health warnings on cigarette packages?	0. No<br>1. Yes<br>3. Did not see any packs of cigarettes	|____|

Section 12: Depression

Knowledge of mental health conditions during **the last four weeks**			
**1201**	In the last four weeks, have you **ever felt exhausted for no good reason?** ?	0. Never<br>1. Rarely<br>2. Sometimes<br>3. Most of the time<br>4. All the time	|____|
**1202**	In the last four weeks, have you ever **felt nervous?** ?	0. Never<br>1. Rarely<br>2. Sometimes<br>3. Most of the time<br>4. All the time	|____|

**1203**	In the last four weeks, have you ever **felt so much nervousness that nothing could calm you down?**	0. Never<br>1. Rarely<br>2. Sometimes<br>3. Most of the time<br>4. All the time	|_____|
**1204**	In the last four weeks, have you **ever felt hopeless** ?	0. Never<br>1. Rarely<br>2. Sometimes<br>3. Most of the time<br>4. All the time	|_____|
**1205**	In the last four weeks, have you ever **felt restless** ?	0. Never<br>1. Rarely<br>2. Sometimes<br>3. Most of the time<br>4. All the time	|_____|
**1206**	In the last four weeks, have you ever felt **so much excitement that you could not hold on?**	0. Never<br>1. Rarely<br>2. Sometimes<br>3. Most of the time<br>4. All the time	|_____|

**1207**	In the last four weeks, have **you ever felt depressed** ?	0. Never<br>1. Rarely<br>2. Sometimes<br>3. Most of the time<br>4. All the time	|____|
**1208**	In the last four weeks, have you ever **felt like you were** trying to do **everything?** ?	0. Never<br>1. Rarely<br>2. Sometimes<br>3. Most of the time<br>4. All the time	|____|
**1209**	In the last four weeks, have you ever **felt such sadness that nothing could cheer you up?**	0. Never<br>1. Rarely<br>2. Sometimes<br>3. Most of the time<br>4. All the time	|____|
**1209**	In the last four weeks, have you ever **felt good for nothing?** ?	0. Never<br>1. Rarely<br>2. Sometimes<br>3. Most of the time<br>4. All the time	|____|

How you've felt for the **past four weeks**.			
If you answered **"Never" to the previous ten questions,** you do not need to answer these additional questions.			
**1211**	During the last four weeks, how many days have you been TOTALLY UNABLE to work, study or go about your daily activities because of these discomforts?	|_________| (Number of days)	
**1212**	[Outside of those days], during the last 4 weeks, HOW MANY DAYS have you been able to work, study or go about your daily activities by REDUCING these activities because of these discomforts?	|_________| (Number of days)	
**1213**	In the last 4 weeks, how many times have you consulted a doctor or other health professional regarding these discomforts?	|_________| (Number of consultations)	
**1214**	During the last 4 weeks, have you ever had physical health problems being the main cause of these discomforts?	0. Never<br>1. Rarely<br>2. Sometimes<br>3. Most of the time<br>4. All the time	|____|

Section 13: Access to Care

<table>
<tr><td>1301</td><td>When was the last time you went to a health facility?</td><td>0. < a week
1. Between a week and a month
2. Between one month and 3 months
3. Between 3 months and 6 months
4. > 6 months
88. Does not remember
99. No answer</td><td>|___|</td></tr>
<tr><td>1302</td><td>Where do you consult when you have a health problem?</td><td>0. CHU or CHR
1. In a private clinic in my neighborhood
2. In a private clinic outside the neighborhood
3. In a medico-social center in my neighborhood
4. In a medico-social center outside my neighborhood
5. I do not consult and buy the drugs at the market/pharmacy
6. Other,
specify:____________________________________
99. No answer</td><td>|___|</td></tr>
</table>

**1303**	Do you have or know a health professional that you see when you have a health problem?	0. No<br>1. Yes, a doctor<br>2. Yes a nurse<br>3. Yes, I don't know its title	|___|
**1304**	Have you told your doctor or healthcare professional that you are a sex worker?	0. No<br>1. Yes<br>88. Does not remember<br>99. No answer	|___|
**1305**	Why didn't you tell them you are a sex worker?	____<br><br>____	
**1306**	Has s/he ever offered to do the STI tests?	a. Syphilis 0. No 1. Yes<br>b. Chlamydia 0. No 1. Yes<br>c. Gonococcal disease 0. No 1. Yes<br>d. Hepatitis B 0. No 1. Yes<br>e. Condylome 0. No 1. Yes<br>f. Hepatitis C 0. No 1. Yes<br>g. HIV 0. No 1. Yes<br>h. Other 0. No 1. Yes<br>Specify: ________________________________	|___| |___| |___| |___| |___| |___| |___| <br>|___|

		99. No answer 0. No 1. Yes	

Section 14: HIV Testing and Care

**1401**	Do you know of a place where you could get tested for HIV?	0. No<br>1. Yes	|___|
**1402**	Have you ever been tested for HIV?<br>*Do not ask any questions about the test results.*	0. No Ú **1406**<br>1. Yes<br>99. Does not wish to reply **Ú 1406**	|___|
**1403**	When did you last get tested?<br>*Only one possible answer.*	1. When donating blood<br>2. After an unprotected report<br>3. During a free and anonymous screening campaign<br>4. Voluntary decision<br>5. During a medical follow-up<br>6. On the proposal (after awareness- raising) of medical staff<br>7. Following an awareness-raising action	|___|

		8. Other<br>Specify:..	
**1404**	When was your last test?<br><br>*If refusal to answer, circle 99.*	1. Less than 3 months<br>2. Between 3 and 6 months<br>3. Between 6 and 12 months<br>4. More than a year<br>96. Don't remember it anymore<br>99. Does not wish to reply	|___|
**1405**	Have you gone back to get your test results?<br>*Do not ask any questions about the test results.*	0. No<br>1. Yes<br>99. Does not wish to reply	|___|
**1406**	Are you aware of the existence of treatment for people who have the AIDS virus (HIV)?	0. No<br>1. Yes	|___|
**1407**	Do you know the HIV status of your regular partner?<br>*If multiple partners, ask the question for the primary partner.*	0. No<br>1. Yes	|___|

**1408**	Would you agree to be tested for HIV	0. No<br>1. Yes	|___|
**1409**	If so, would you like to know the result of your HIV test?	0. No<br>1. Yes	|___|
**1410**	If not, why not?	____<br>____	

nb:

1- Check that all questions are filled in

2- Thank the respondent

3- Invite the respondent to go to the collection room by giving him a coupon
